# Adaptive PEG Bis-dendron Hydrogels with Tunable Mechanics and Bioactivity

**DOI:** 10.1021/acs.chemmater.6c00419

**Published:** 2026-04-15

**Authors:** Evgeny Apartsin, Noël Richard, Birgit Habenstein, Antoine Loquet, Sophie Lecomte, Marie-Christine Durrieu

**Affiliations:** 1 Univ. Bordeaux, CNRS, Bordeaux INP, CBMN, UMR 5248, Pessac F-33600, France; 2 Univ. Poitiers, CNRS, XLIM, UMR 7252, Futuroscope Chasseneuil F-86360, France; 3 Univ. Bordeaux, CNRS, Bordeaux INP, CBMN, UMR 5248, IECB, Pessac F-33600, France; 4 Univ. Bordeaux, CNRS, INSERM, IECB, US1, UAR 3033, Pessac F-33600, France

## Abstract

Most organoids are cultured in Matrigel, a complex and poorly defined matrix that limits our mechanistic understanding. Synthetic hydrogels offer a versatile alternative, providing precise control over mechanical and biochemical cues. Using two topologically different types of hydrogel precursors, branched poly­(ethylene glycol) (PEG) and PEG bisdendrons, we have obtained a library of hydrogels via thiol–ene cross-linking with branched PEG-thiol. Their chemical conversion was monitored by Raman spectroscopy, while swelling and mechanical properties, including elastic, viscoelastic, and relaxation parameters, were systematically evaluated. Bisdendron hydrogels dissipate stress through abundant weak interactions, conferring adaptive viscoelastic behavior, an underexplored feature in a 3D culture. To link macromolecular dynamics with bulk properties, polymer chain mobility and internal architecture were probed using MAS solid-state NMR and freeze-fracture cryo-SEM. To introduce bioactivity, RGD peptides were used and immobilized via thiol–ene chemistry, forming spatially organized clusters within the hydrogels. This strategy enables the design of customizable matrices with tunable mechanics, adjustable porosity, and controlled bioactive presentation, closely mimicking native microenvironments. Our platform can provide a chemically defined and versatile toolbox for organoid culturing.

## Graphical abstract

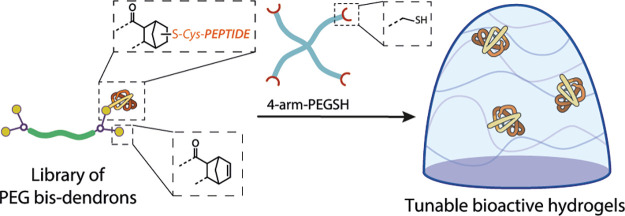

## Introduction

Recent advances in three-dimensional (3D) culture systems, such as spheroids, organoids, and tumoroids, have transformed applied research by providing models that more accurately reproduce the architecture and microenvironment of living tissues and tumors compared to traditional two-dimensional cultures.^1–3^ These systems enhance predictive power in drug screening, disease modeling, and personalized medicine by recapitulating key features such as cell–cell and cell–matrix interactions, diffusion gradients, and physiological heterogeneity.

The effective culture of these 3D assemblies depends on their integration into a suitable extracellular matrix (ECM) that supports adhesion, differentiation, and tissue-specific organization. Matrigel, the current commercial gold standard, is widely used due to its biochemical richness but suffers from significant drawbacks, including undefined composition, batch-to-batch variability in protein and growth factor content, mechanical property inconsistencies, and safety concerns related to animal origin and potential contaminants.^4,5^ These limitations compromise reproducibility and translational applicability, thus showing the need for rationally designed and tunable hydrogel platforms that can reliably support the growth and function of complex 3D cell assemblies.

Hydrogels based on natural polymers (gelatin, collagen, alginate, etc.) create a natural-like chemical environment,^6–9^ but the physicochemical and mechanical properties of these polymers are difficult to manipulate within a single formulation. Synthetic hydrogels can overcome these problems and greatly reduce the amount of batch-to-batch variation.^10,11^ The ability to modulate the chemical structure of synthetic hydrogels makes it possible to finely tune the mechanical and biochemical properties of a hydrogel scaffold depending on the application.^12–19^ Among synthetic polymers, poly­(ethylene glycol) (PEG)-based hydrogels are of particular interest due to their bioinertness, well-defined molecular weight-dependent properties, and synthetic convenience. The multivalency of star-shaped PEG (4-arm and 8-arm branched PEG species) makes it an attractive option for hydrogel development.^20–22^ R. Cruz-Acuña et al. present a protocol to generate human intestinal and lung organoids using a synthetic, chemically defined star-shaped PEG-based hydrogel scaffold.^23,24^ PEG polymers bearing photo-cross-linkable units (acrylate, allyl/vinyl, norbornene) are convenient precursors for hydrogels, as they can be quickly (co)­polymerized into a 3D scaffold.^25,26^ In particular, branched PEG-norbornene derivatives have been used as scaffold precursors for cell cultivation for more than a decade, yielding hydrogels with elasticities in the range 1–3 kPa.^27–30^

Dendrimers, a special family of branched molecules, have been widely explored as precursors for hydrogels^31^ due to their branched, multifunctional structures, tunable surface chemistry, and ability to form chemically^32,33^ and physically cross-linked^34,35^ networks. The dense and well-defined architecture of dendrimers provides a high density of surface functional groups, enabling precise functionalization for cell adhesion ligands, growth factor immobilization, or incorporation of other bioactive molecules. This versatility facilitates the creation of hydrogels with tailored mechanical strength, degradation profiles, and biological activity, allowing them to mimic native extracellular matrices more effectively and support the growth, proliferation, and differentiation of spheroids, organoids, and tumoroids.^36^

In particular, bisdendrons hold great potential for the preparation of materials for biomedical applications. A recent example, reported by the Malkoch group, is an artificial ECM for skin and bone regeneration, consisting of an allyl-terminated bis-MPA-PEG bisdendron cross-linked with PEG dithiol and a natural polymer blend obtained from the decellularized placenta.^37^ The placenta-derived blend is meant to provide chemical and biochemical cues for tissue regeneration, whereas the bisdendron is responsible for the overall material stiffness. This formulation exhibited considerably high elasticity after cross-linking (*G*′ = 1–52 kPa), efficiently supported Raw 264.7 cell growth, and was compatible with bioprinting technology as a precursor solution.

In this work, we report the design and development of bioactive hydrogels based on original PEG-norbornene bisdendrons. The bisdendron topology permits the organization of norbornene units into low-generation dendritic clusters, which allows not only to precisely tune the mechanical properties but also to control the hydrogel porosity and bioactivity through the immobilization of application-specific biomolecular cues. Our goal is to establish a chemically defined hydrogel platform with tunable elasticity, viscoelasticity, porosity, and controllable bioactive functionalization as key design parameters for the rational design of hydrogels as artificial extracellular matrices.

## Results and Discussion

To assemble hydrogel networks that can serve as artificial ECMs for cell culture, we chose a strategy based on the on-demand photoinduced cross-linking between norbornene fragments and thiol groups. This process, referred to as thiol–ene chemistry, has been demonstrated to yield biocompatible hydrogels.^38,39^ The gelation kinetics are faster than for other cross-linking groups,^40^ making it an attractive option for hydrogel bioprinting. Using thiol–ene chemistry, it is also possible to incorporate bioactive peptides during hydrogel polymerization.^22^ Furthermore, norbornene clusters can create peptide-rich islands in the hydrogel volume, which is known to enhance cell-material interaction through multisite recognition of biochemical signals by surface receptors.^41^

As precursors for hydrogel formation, we chose two types of norbornene-bearing PEG polymers: a commercial branched-chain, 4-arm-PEG-NB that is known to be compatible with cell culture,^42^ and two series of bisdendrons based on linear PEG polymers bearing first-generation norbornene-terminated dendritic branches. To form hydrogels, norbornene-containing precursors were cross-linked with a branched PEG polymer, 4-arm-PEG-SH, bearing thiol groups at the extremities. Alternatively, a bioactive peptide was grafted to PEG-norbornene precursors prior to cross-linking, and then, a hydrogel was formed (Figure 1).

**Figure 1:**
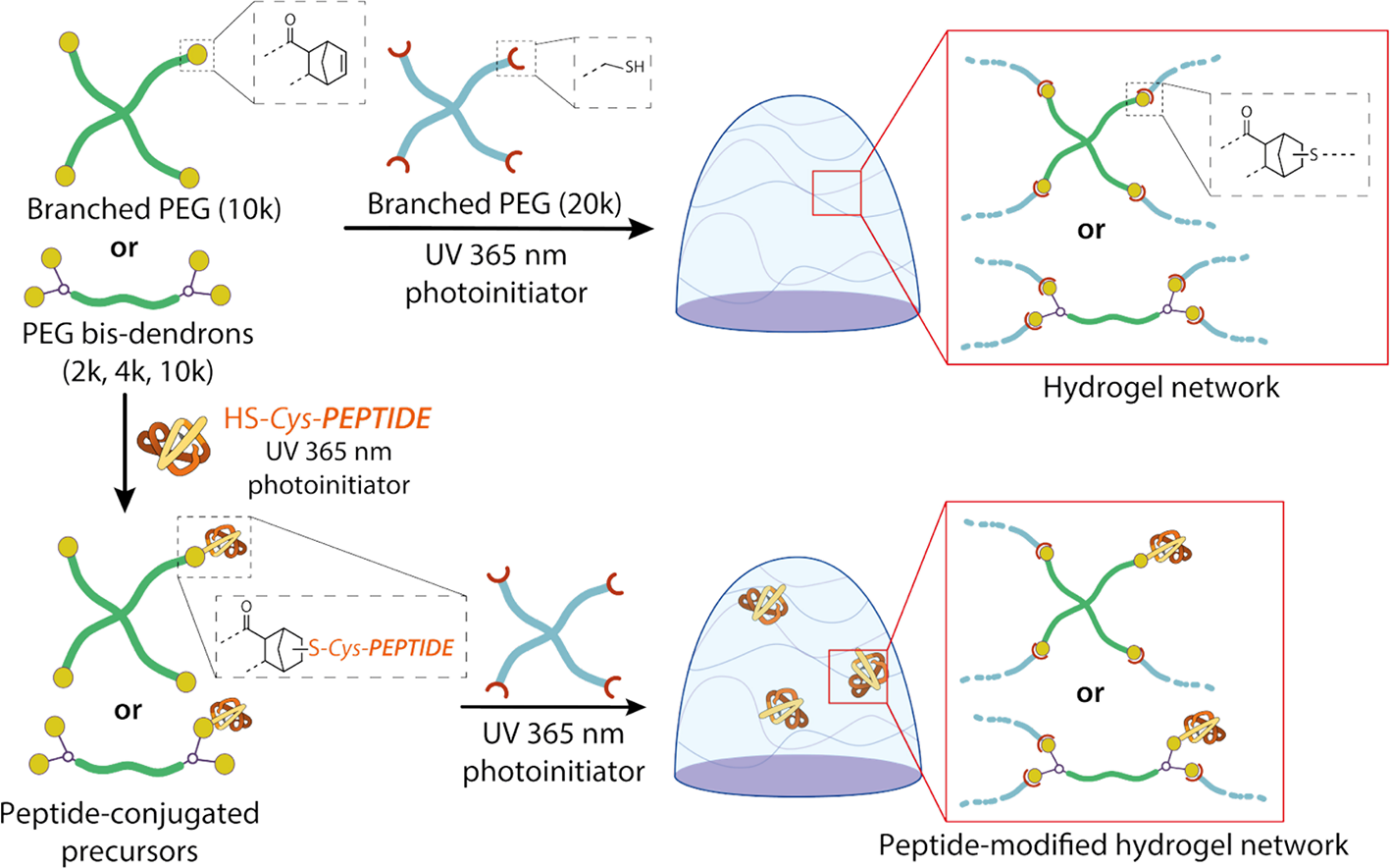
Scheme of cross-linking between PEG-norbornene and PEG-thiol polymers to form a hydrogel network.

### Synthesis of PEG-Norbornene Bisdendrons

Bisdendrons based on linear PEG were designed to bear branching units at the extremities, with two norbornene units being grafted to each of them. Such a design permits conservation of the norbornene: thiol stoichiometry during cross-linking, however drastically changing the distribution of norbornene units, in comparison with the branched PEG-norbornene precursor.

To introduce norbornene units onto the PEG extremities, two types of reactive functional blocks were used: norbornene dicarboxylic anhydride (**1**) and norbornene dicarboximidohexanoic acid (**2**) obtained from (**1**) by reaction with 6-aminohexanoic acid (Scheme 1).

**Scheme 1:**
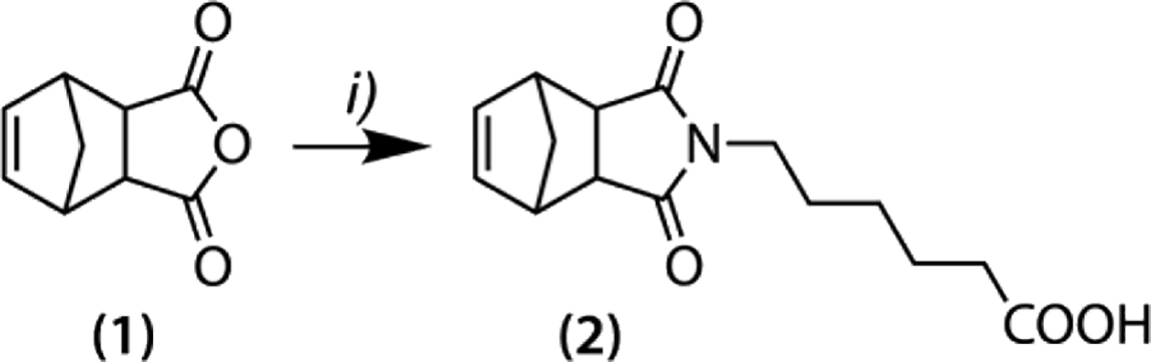
Reactive Norbornene Functional Blocks^a^

a Conditions: (i) H_2_N­(CH_2_)_5_COOH, AcOH, 110 °C, 85%.

To obtain PEG bisdendrons, linear PEG (MW 2000, 4000, or 10,000) was first activated through tosylation, and subsequently a branching unit, dimethyl 5-hydroxyisophthalate was grafted. Dimethyl 5-hydroxyisophthalate is known as a convenient branching unit compatible with iterative grafting,^43^ and it has been recently used for building dendrimers and dendrons as therapeutic agents^44^ and molecular recognition elements.^45^

Ester-terminated synthon (**5**) was further reduced with borane-dimethylsulfide complex to yield benzyl alcohol derivative (**6**), which was further functionalized with norbornene functional blocks (**1**) or (**2**) to obtain norbornene-terminated bisdendrons with anionic surface groups (norbornene carboxylate) referred thereafter to as 2kNBA, 4kNBA, 10kNBA (**7a**–**c**), or neutral surface groups (norbornene carboxamide) referred thereafter to as 2kNB, 4kNB, and 10kNB (**8a**–**c**), as shown in Scheme 2. The completeness of norbornene grafting was confirmed by the disappearance of benzyl alcohol signals in NMR spectra (see pp. S10–S11 in the Supporting Information).

**Scheme 2:**
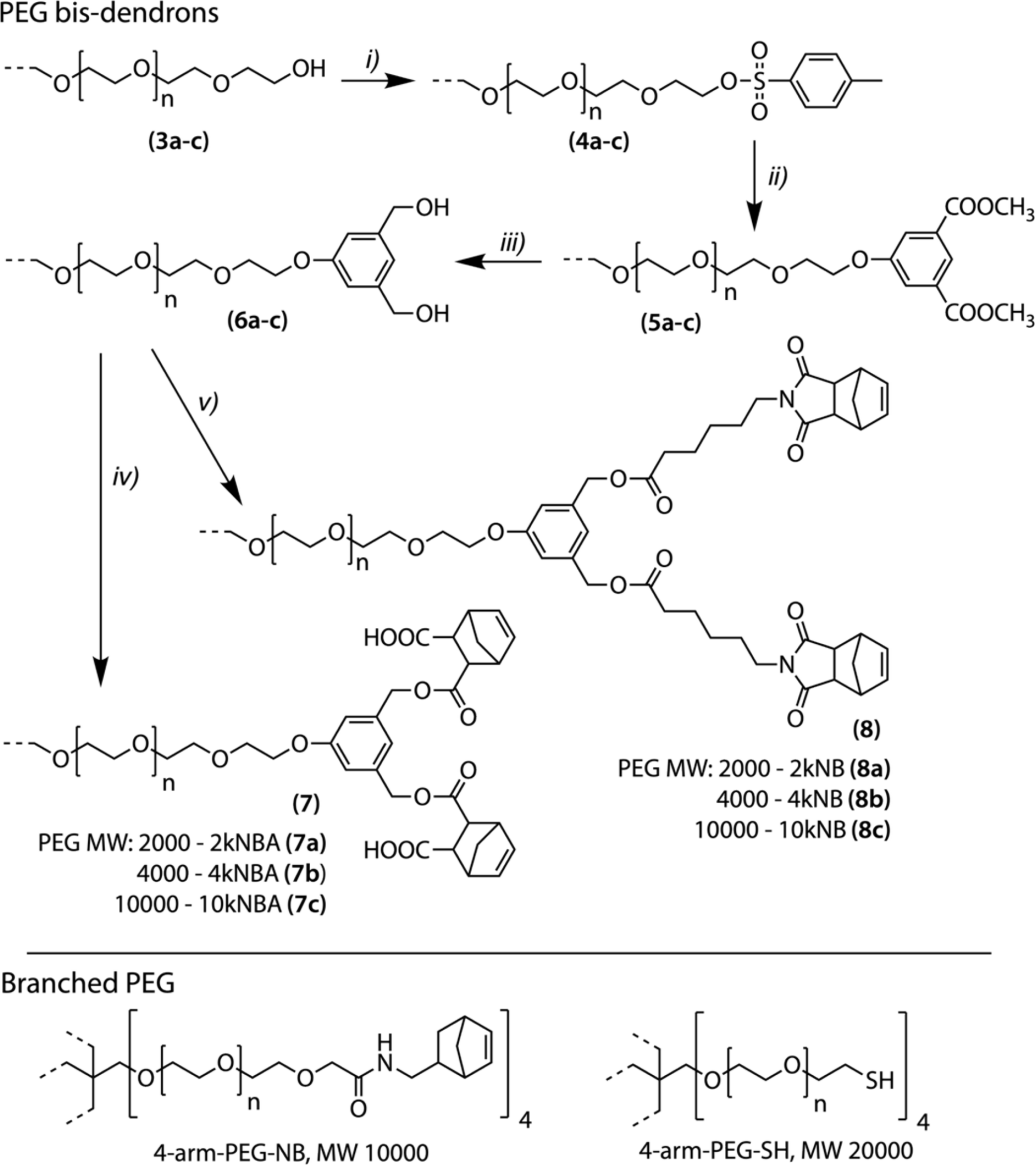
Synthesis of PEG-Norbornene Bis-Dendrons (**7**), (**8**); Commercial Branched PEG precursors^a^

a Conditions: (i) TsCl, NEt_3_, CH_2_Cl_2_, 0 °C, 87–94%; (ii) dimethyl 5-hydroxyisophthalate, K_2_CO_3_, CH_3_CN, 85 °C, 65–96%; (iii) BH_3_•(CH_3_)_2_S, THF, 80 °C, 98%; (iv) (1), NEt_3_, CH_2_Cl_2_, 0 °C, 76–96%; (v) (2), (COCl)_2_, NEt_3_, CH_2_Cl_2_, 0 °C, 95–96%.

At each step of modification, the resulting PEG products were precipitated in diethyl ether to remove ether-soluble impurities and, importantly, to obtain polymers as powder-like solids easy to manipulate and stable upon storage. As it was reported for PEG-based polymers, the longer polymers are more efficiently extracted from water-organic mixtures and better precipitated in diethyl ether.^46^

### Formation of Hydrogel Networks

Herein, we report a novel approach to obtain soft materials (G′ < 10 kPa) based on PEG bisdendrons. To achieve this, we synthesized precursors with PEG chains of various lengths and small dendron units bearing reactive norbornene functionalities at the extremities. The hydrogel materials were obtained by UV-induced cross-linking between a PEG-norbornene polymer and the branched thiol-terminated polymer 4-arm-PEG-SH, with a total PEG concentration of 10% or 5% maintained for PEG-norbornene precursors of different molecular weights. PEG precursors and a photoinitiator (LAP) were mixed in PBS at a norbornene:thiol molar ratio of 1:1. The properties of PEG bisdendrons-containing hydrogels were compared with those based on a branched 4-arm-PEG-norbornene polymer.

Upon cross-linking, thiol groups are converted into thiol radicals that are grafted to C=C bonds in norbornene units to form thioethers (Figure 1). Ideally, both groups react stoichiometrically and are consumed simultaneously. To probe the conversion of reactive groups upon the formation of hydrogels, we employed Raman spectroscopy (Figure 2). In norbornene units, a characteristic band is observed at 1580 cm^–1^ corresponding to the C=C stretching mode.^47^ This band is meant to disappear upon conversion into a thioether, thus being a convenient signal for monitoring the completeness of the reaction. The band at 1770 cm^–1^ assigned to the C=O stretching mode is not supposed to change in the reaction and therefore can be used as a reference. In the PEG-thiol, there is a characteristic band at 2570 cm^–1^ assigned to the S–H stretching mode^48^ and supposed to disappear upon hydrogel formation. It should be noted that the concentration of these groups in samples is quite low in comparison with the ethylene glycol units in PEG, and as a consequence, their bands are not intense even in the spectra of precursors. Nevertheless, the consumption of the norbornene C=C bond upon the reaction with thiols is visible in Raman spectra. The intensity of the reference C=O band is greatly decreased due to the dilution of norbornene with high-molecular-weight PEG in a hydrogel; however, this band is still visible. Thiol groups appear to be mostly consumed. Thus, the gelation is complete, except for a tiny excess of free thiols (<2% according to the Ellman’s test, see Table S1) still present in the material.

**Figure 2:**
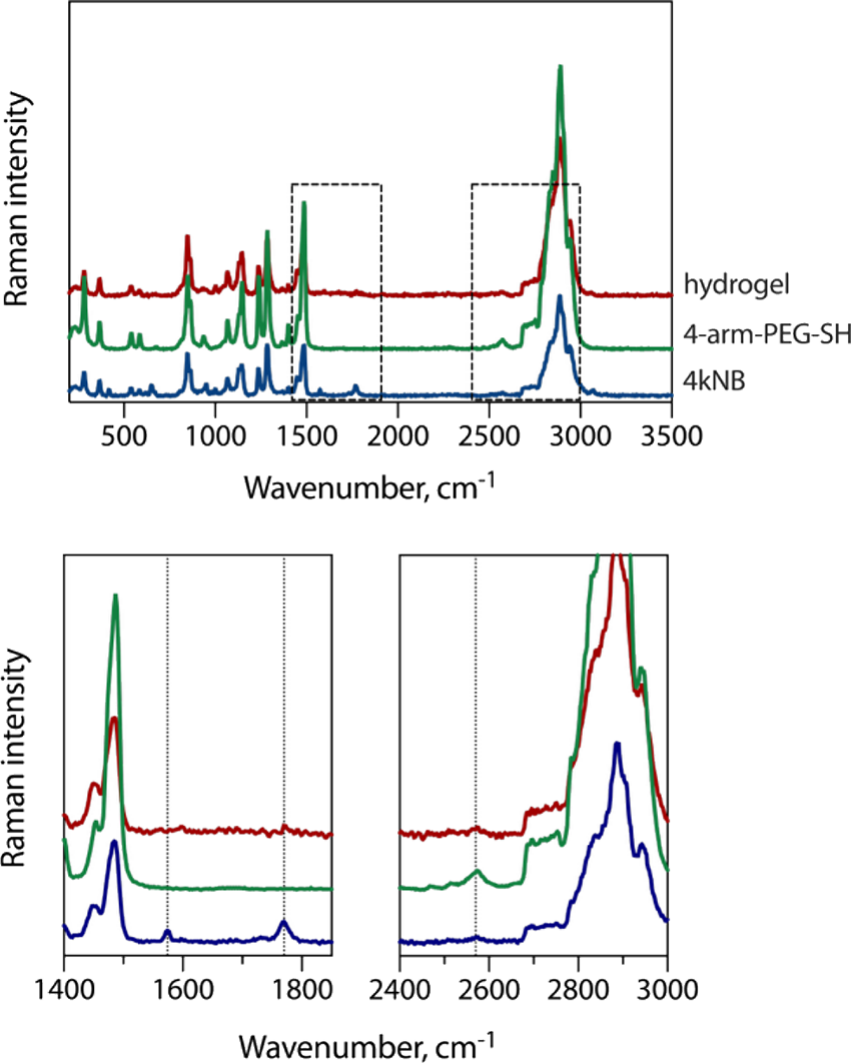
Raman spectra of the PEG-norbornene precursor 4kNB, PEG-thiol precursor 4-arm-PEG-SH, and their hydrogel (λ_ex_ = 532 nm). Selected zones (dashed rectangles) are zoomed below. Dotted lines show the C=C stretching mode (1574 cm^–1^) and C=O stretching mode (1770 cm^–1^) in norbornene groups as well as the S–H stretching mode (2570 cm^–1^) in 4-arm-PEG-SH.

The materials obtained exhibited significant swelling in PBS, and the swelling ratio and material volume increase were assessed (Table 1). The volume stretch can serve as a convenient measure of hydrogel swelling,^49^ and it also permits the estimation of the apparent concentration of components in the swollen material.

**Table 1:** Swelling Parameters of PEG-Norbornene-Based Hydrogels^a^

PEG-norbornene precursor	swelling ratio (g/g)	volume stretch (v/v)
4-arm-PEG-NB	21.6 ± 0.7	2.2 ± 0.2
2kNBA (7a)	16.3 ± 0.8	3.3 ± 0.3
4kNBA (7b)	27.5 ± 1.2	4.2 ± 0.4
10kNBA (7c)^b^	39.9 ± 2.1	5.4 ± 0.3
2kNB (8a)^c^		
4kNB (8b)	16.5 ± 1.0	3.1 ± 0.2
10kNB (8c)^b^	32.2 ± 1.5	5.9 ± 0.4

a PEG-norbornene precursors were cross-linked with 4-arm-PEG-SH at 10% total PEG, norbornene:thiol ratio 1:1, unless otherwise stated.

b Solid materials have been obtained at the norbornene:thiol ratio of 2:1.

c Incomplete gelation.

Materials containing 4-arm-PEG-NB showed a 1.8-fold volume increase with a swelling ratio of ∼20, while those containing linear 10kPEG bisdendron precursors undergo a 6-fold volume increase (swelling ratio ca. 40 for 10kNBA and ca. 32 for 10kNB). These drastic differences can be attributed to the denser cross-linking between 4-arm-PEG-NB and 4-arm-PEG-SH precursors (i.e., shorter polymer chain lengths between cross-links) compared to the cross-linking of a 4-arm-PEG-SH with a PEG bisdendron. The swelling values gradually decrease in the row 10*k* > 4k > 2k (Table 1).

Representative swelling kinetics of the different PEG-norbornene hydrogels in PBS are shown in Figure S2, indicating a rapid initial uptake of water followed by a plateau consistent with equilibrium swelling within the experimental time frame. At a fixed total PEG content, bisdendron-based networks reach higher equilibrium swelling than the 4-arm-PEG-NB hydrogel, reflecting the lower cross-linking density and longer chains between cross-links in the former.

Comparison of the anionic (NBA) and neutral (NB) bisdendron series reveals that the surface charge further modulates the equilibrium swelling ratio. For a given PEG chain length, NBA hydrogels display a slightly higher volume stretch than their NB analogues, consistent with additional water uptake driven by hydration of the carboxylate-terminated dendritic units. This effect becomes particularly evident for long-chain 10k bisdendrons, where the combination of low cross-linking density and charged termini yields the highest swelling values within the library. The highly hydrated state suggests the presence of larger pores within the material,^50^ which can enhance the diffusion of nutrients and metabolites to and from cells encapsulated in the hydrogel.

Li et al. reported swelling to correlate with the density of cross-linking in PEG-based hydrogels.^46^ Comparing materials prepared via chain-growth polymerization (PEGDA hydrogels) and step-growth cross-linking between branched PEG-NB and PEG-SH, they found that the materials prepared by chain-growth polymerization were swollen more abundantly than step-growth cross-linked materials. This, in turn, correlated with the apparent pore size in the hydrogel, which has been proven by the protein diffusion studies in hydrogels. The nature of these differences is in the cross-linking density expressed as the molecular weight of a chain between cross-links. In PEG-based hydrogels, the networks formed by monomers with larger molecular weight have lower cross-linking density and longer PEG chains between cross-links, and the looser network has a higher capacity for water uptake and swelling.^49^ In addition, the presence of pendant polymer chains can increase the hydrogel swelling rate.^51^ This might be the case for our hydrogels containing 10kPEG bisdendrons, where the concentration of reactive groups in an elementary reaction volume is relatively low, especially at the end of cross-linking.

### Mechanical Properties of Hydrogels

In designing hydrogel materials to support the development of spheroids, organoids, and tumoroids, we aimed for the elasticity values in the range of *G*′ = 0.1–10 kPa, known to cover most parts of ECM elasticities in human tissues.^52^ To modulate the elasticity, we changed the molarity and topology of the PEG-norbornene precursor as well as the total concentration of PEG.

The mechanical properties of PEG-norbornene materials were first probed by strain sweep oscillatory rheology. For all materials under study, a linear viscoelastic (LVE) region was observed in the rheology profiles. The LVE region was found to be at 0.1–10% strain for 4-arm-PEG-NB, 0.1–30% strain for 2kPEG, 0.5–30% strain for 4kPEG, and 1–100% strain for 10kPEG bisdendrons (Figure S3). In 10kPEG-based gels, the LVE region is difficult to deduce at small strains (0.1–1%) due to the dispersion of storage and loss moduli values. These findings suggest that the use of bisdendrons as precursors permits extending the LVE region toward bigger strains, which in turn shows higher material compliance.^53^

Having chosen the strain of 3% to remain in the LVE region, we assessed frequency sweeps for the hydrogels (Figure 3, Figure S4, and Table 2). At 10% PEG, 4-arm-PEG-NB forms a material with a *G*′ of 2.8 kPa, while PEG bisdendron 2kNBA yields an even stiffer material with a *G*′ of 3.3 kPa. PEG bisdendrons 4kNBA and 4kNB form gels with close elasticity values of *G*′ ≈ 700 Pa. 10kPEG bisdendrons produce very soft gels of *G*′ ≈ 60–170 Pa. The observed differences in elastic modulus can be directly associated with changes in network architecture, i.e., in the effective cross-linking density and the molecular weight of PEG chains between cross-links. Interestingly, its uncharged analog 2kNB forms a heterogeneous gel (with a *G*′ of 58 Pa) likely due to the imperfect solubility of the precursor in PBS used for the hydrogels preparation. However, when dissolved in pure deionized water, 2kNB gives a clear solution. This finding can be explained by the backfolding of norbornene-terminated branches and their screening by the PEG fragments at physiological ionic strength, which makes norbornene units poorly accessible for the cross-linking.

**Figure 3:**
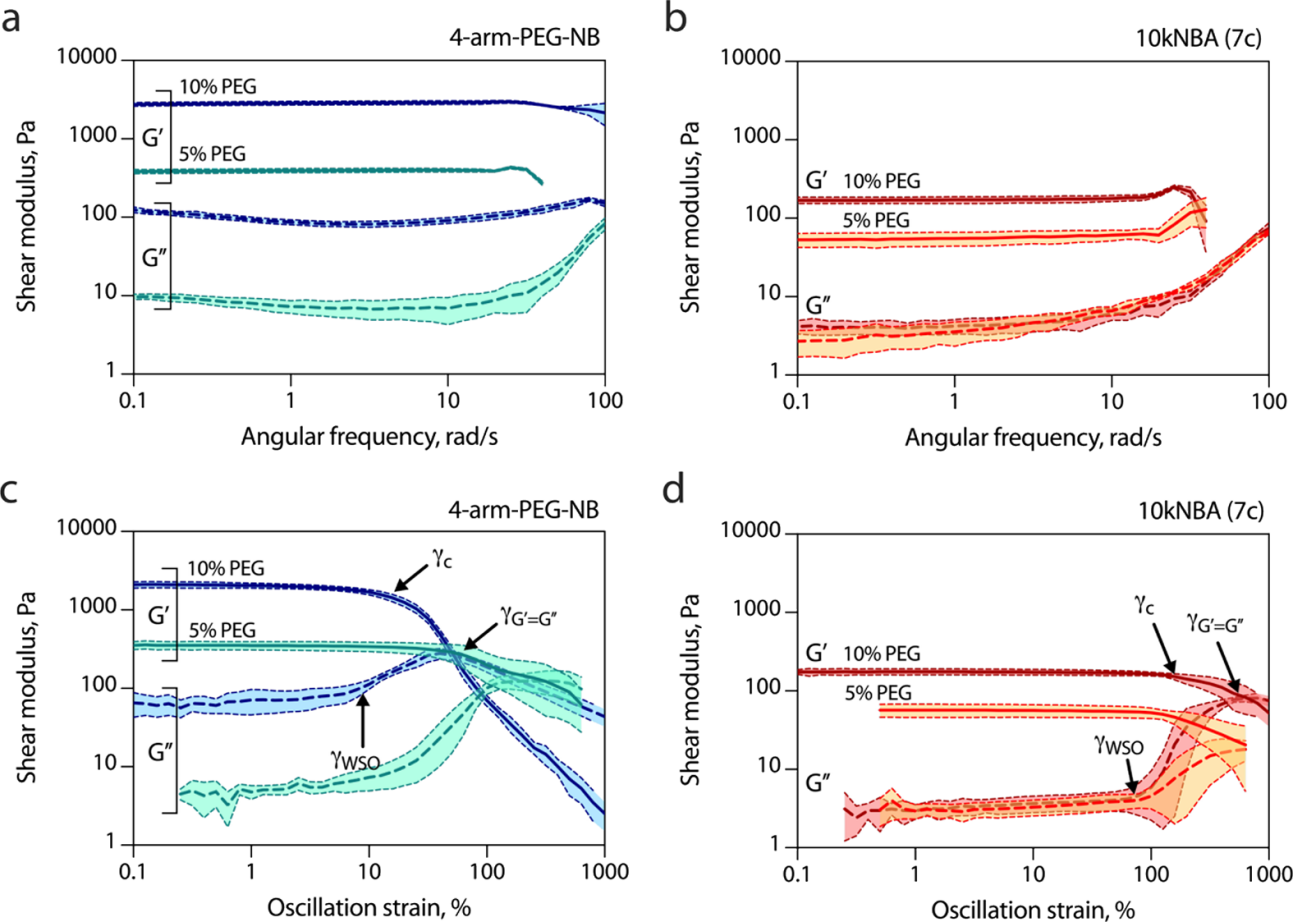
Representative oscillatory shear rheology profiles for hydrogels prepared from 4-arm-PEG-NB (a, c) or 10kNBA PEG-norbornene bisdendrons (b, d) and 4-arm-PEG-SH precursors. (a,b) Frequency sweeps, oscillation strain: 3%. (b, d) Strain sweeps, oscillation frequency 10 rad/s. Data presented as mean ± s.d. (*n* = 4–5).

**Table 2:** Elastic, Viscoelastic, and Relaxation Parameters of PEG-Norbornene-Based Hydrogels^a^

PEG-norbornene precursor	*G*′ (Pa)	*G*″ (Pa)	relaxation (%)	apparent τ_1/2_ (s)
**10% Total PEG**				
4-arm-PEG-NB	2778 ± 76	86 ± 6	18 ± 4	123 ± 9
2kNBA (**7a**)	3320 ± 210	52 ± 4	20 ± 2	48 ± 4
4kNBA (**7b**)	660 ± 50	4.0 ± 0.3	15 ± 1	673 ± 35
10kNBA (**7c**)^b^	170 ± 15	1.3 ± 1.3	13 ± 1	179 ± 11
2kNB (**8a**)	58 ± 5	3.0 ± 0.2	–^c^	–^c^
4kNB (**8b**)	713 ± 55	14 ± 1	28 ± 2	104 ± 8
10kNB (**8c**)^b^	16 ± 1.3	–^c^	22 ± 2	168 ± 12
**5% Total PEG**				
4-arm-PEG-NB	393 ± 24	7.3 ± 1.3	20 ± 2	71 ± 5
2kNBA (**7a**)	456 ± 35	4.0 ± 0.4	8 ± 1	20 ± 2
4kNBA (**7b**)	205 ± 18	2.9 ± 0.3	5 ± 0.5	116 ± 9
10kNBA (**7c**)^b^	55.3 ± 11.1	3.6 ± 1.2	1 ± 0.2	–^c^
2kNB (**8a**)	127 ± 10	2.3 ± 0.2	4 ± 0.5	7 ± 0.8
4kNB (**8b**)	306 ± 25	1.5 ± 0.1	9 ± 1	80 ± 6
10kNB (**8c**)^d^				

a PEG-norbornene precursors were cross-linked with 4-arm-PEG-SH at a norbornene:thiol ratio of 1:1, unless otherwise stated.

b Solid materials have been obtained at the norbornene:thiol ratio of 2:1.

c Value could not be measured.

d Incomplete gelation.

The viscoelasticity profiles generally follow the same trend as the elasticity ones. However, the *G*″ values remain consistently low (1–50 Pa, tanδ ≈ 0.02 in all cases), indicating a minimal viscoelastic contribution of the material modulus. At 5% PEG, the elasticity values fall within the range 100–500 Pa for both branched PEG and bisdendrons. In contrast, the viscoelasticity profiles reveal a clear distinction between the two topologies: the 4-arm-PEG-NB-based material has a *G*″ value of 22 Pa, with a tan δ value reaching 0.05, indicating a higher viscoelastic component in comparison with other cases. The materials formed by PEG bisdendrons show *G*″ values in the range of 1.5–4 Pa.

The *G*″ (1–50 Pa) and tanδ values (0.02–0.05) are quite typical for the PEG hydrogel materials currently used for cell cultivation.^39,54,55^ However, although viscoelasticity is considered crucial for the development of various cell types, in the case of tumor spheroids, its role is not yet universally agreed upon. For this reason, a series of materials with similar chemical properties and structures but having different viscoelasticity values can help to conclude the appropriateness of the material viscoelasticity for organoid culture.

It is worth noting that the behavior of the *G*″ profiles (Figure 3 and Figure S4) indicates differences in the viscoelastic behavior in the hydrogels based on branched PEG and PEG bisdendrons. At high frequency, the materials show a continuous increase in *G*″ values in comparison with the linear viscoelastic region. However, in the 4-arm-PEG-NB-based material, an ∼3-fold increase was observed, whereas in the bisdendron-based materials, a 10-fold to 100-fold increase was found, with longer PEG bisdendrons showing a higher increase. These differences were observed both at 10% and 5% total PEG. This is evidence of the increased mobility of polymer chains and subsequently, of the mobility-associated friction between polymer chains and the surrounding buffer contributing to the energy dissipation in hydrogels.^56,57^ The fact that the observed effects become more pronounced with the increase of the molecular weight of PEG bisdendrons suggests that the interchain friction is impacted by the molecular weight of the polymer between cross-links.

Cells embedded in 3D matrices routinely generate deformations that extend beyond the small-amplitude linear viscoelastic regime, for example, during collective migration, matrix compaction, and remodeling, or under external mechanical loading in bioreactors.^58–60^ Consequently, characterizing material behavior at larger strains beyond the LVE region provides access to mechanical thresholds that are directly relevant for long-term 3D culture, handling, and device-level perturbations, rather than describing only infinitesimal deformations.

A characteristic measure of the material behavior is the critical strain (γ_c_), the strain value separating linear and nonlinear viscoelasticity regions. In amplitude sweep profiles, *G*′ begins to decrease above γ_c_ (irreversible network rearrangements and softening) due to the shear thinning of the material^61^ (Figure 3c,d and Figure S3), evidencing the inelastic energy dissipation.^62^ Comparing γ_c_ values for materials based on branched PEG and PEG bisdendrons (Table 3), we observe sharp differences between the three types of polymer precursors used. In the 4-arm-PEG-NB-based hydrogel, the critical strain value has been found to be as high as 16%, whereas in the 2kNBA-based hydrogel, having comparable *G*′, the critical strain value has appeared to be considerably higher (134%). In turn, in the hydrogels based on the PEG bisdendron precursors with the same molar weight as 4-arm-PEG-NB, i.e., 10kNBA and 10kNB, the critical strain value appeared to be an order of magnitude higher than for the branched PEG (Table 3). Interestingly, the hydrogel based on neutral norbornene-containing precursor 4kNB behaves not like its anionic analogue 4kNBA, but rather like 2kNBA containing a shorter PEG chain (see explanation below).

**Table 3:** Critical Strain Parameters of PEG-Norbornene-Based Hydrogels^a^

PEG-norbornene precursor	γ_c_ (%)	γ_WSO_ (%)	γ(*G*′ = *G*″) (%)
4-arm-PEG-NB	21.5 ± 1.4	6.6 ± 1.1	53.3 ± 3.5
2kNBA (**7a**)	134 ± 9	32 ± 2	200 ± 15
4kNBA (**7b**)	296 ± 18	79 ± 4	795 ± 40
10kNBA (**7c**)^b^	113 ± 10	69.3 ± 0.7	508 ± 33
2kNB (**8a**)^c^			
4kNB (**8b**)	137 ± 10	25 ± 2	250 ± 25
10kNB (**8c**)^b^	1000	–^d^	>1000

a PEG-norbornene precursors were cross-linked with 4-arm-PEG-SH at 10% total PEG, a norbornene:thiol ratio of 1:1, unless otherwise stated.

b Solid materials have been obtained at the norbornene:thiol ratio of 2:1.

c Incomplete gelation.

d Value could not be measured.

To explain the differences found, we analyzed the *G*″ profiles (Figure 3 and Figure S3). A weak strain overshoot (WSO, i.e., the G″ increase followed by a sharp decrease upon increasing γ in the nonlinear region)^63,64^ was observed in all hydrogel samples studied. This overshoot reflects transient reinforcement of the network by weak, reversible interactions (e.g., interchain associations, such as van der Waals interactions or hydrogel bonding) to form a weakly (micro)­structured hydrogel that is progressively broken under large deformation.

In the case of PEG-NBA-based hydrogels, the backbone is additionally extended due to the electrostatic repulsion from the charged groups in dendritic parts and also stabilized by intrinsic hydrogel bonding of carboxylic groups (compare profiles for 4kNB and 4kNBA in Figure S3). When an external strain is applied, the hydrogel structure stabilized by weak interaction is able to resist deformation up to a certain strain, where *G*″ increases. Then, the complex structure is destroyed by large deformation over the critical strain, and G″ decreases.^64,65^ The strain at which the WSO regime begins (γ_WSO_) represents the resistance of the polymer network to the stress without the contribution of weak interactions.^66^ Accordingly, comparing values of γ_c_ and γ_WSO_, we can assume the role of polymer chain structuring in energy dissipation upon stress. These effects are the most pronounced when comparing hydrogels based on 4-arm-PEG-NB, 4kNBA, and 4kNB. A drastic difference between γ_c_ and γ_WSO_ (>100%) in materials containing PEG bisdendrons suggests that multiple weak interactions between linear PEG chains greatly stabilize hydrogels toward the external stress. This effect is much less expressed in the hydrogel based on the branched PEG.

The position of the hydrogel transition between solid-like and fluid-like regimes, γ­(*G*′ = *G*″), can also serve as a characteristic measure of the hydrogel properties.^67^ The dynamics of the transition strain values generally follow the regularities found for the γ_c_ and γ_WSO_ (Table 3), thus supporting the conclusion of the weak interaction contribution into the hydrogels' response to the external stress. These parameters delineate the strain amplitudes beyond which the network begins to dissipate energy through irreversible rearrangements or damage, which is informative for predicting how the gels will respond to cell-generated forces and macroscopic manipulations such as pipetting, transport, or compression in culture devices.

Experimental studies on strain overshoot in soft biomaterials (e.g., plantar soft tissue analogs) have further indicated that minimal overshoot values (0.3–0.9%) can induce stress variations up to 25%, directly impacting outcome measures such as cell adhesion, migration, and structural integrity.^68^ Thus, tuning the critical strain (typically within the 10–40% range) and controlling strain overshoot positioning (1–5% for biological matrices) are essential strategies for optimizing biomaterial platforms in advanced tissue engineering and mechanobiology research.

When assessing the stress–relaxation properties of hydrogel materials, we found that the relaxation degree does not exceed 30% within 1500 s of measurement (Table 2 and Figure S5). Hydrogels based on branched PEG-norbornene exhibit a plateau at 20% relaxation for both 10% PEG and 5% PEG. However, at 5% PEG, the relaxation occurs more rapidly with τ_1/2_ being 71 s, compared to 123 s at 10% PEG.

The materials based on PEG bisdendrons behave otherwise: at 10% PEG, they show a similar relaxation degree (15–30%) and generally comparable relaxation time (48–123 s), with the exception of the precursor 4kNBA, which shows a significantly longer relaxation time of 673 s. However, at 5% PEG, the relaxation degree drops to 5–8%, with the relaxation time being of the same order of magnitude as that at 10% PEG (20–120 s).

The observed differences in the behavior of hydrogels permit us to assume that in the latter case, the mechanics of PEG bisdendrons-based hydrogels is surprisingly close to the chain-growth polymerized hydrogels (higher molecular weight of chains between two cross-links, more pronounced entropic effects, abundant weak interactions, higher swelling ratio), which is in agreement with the NMR data.^69^ In branched PEG-based hydrogels, however, the material is an example of the step-growth reaction, where the cross-link density is higher, and the structure is generally more rigid at equal concentration of reactive groups, as observed, for instance, in large strain oscillation shear profiles.^70^

Recently, López-Serrano et al. reported a series of PEGDA-400 and PEGDA-4000 blend hydrogels with tunable elasticity as supports for hMSC adhesion and osteogenic differentiation.^17^ Their data permit us to compare the behavior of PEG hydrogels obtained herein with other PEG hydrogels. PEGDA-400 hydrogels show concentration-dependent elasticity (5–40 kPa), but for all species, the γ_c_ value was <1%, thus indicating the low contribution of intermolecular forces to the energy dissipation. The PEGDA-400/PEGDA-4000 20/80 blend shows a *G*′ of 3 kPa, which is close to the values we obtained for the 2kNBA-based hydrogel; however, this material also had γ_c_ < 1%. In contrast, the PEGDA-4000 hydrogel had *G*′ ≈ 700 Pa, which corresponds to our findings for the 4kPEG hydrogels, and γ_c_ ≈ 7%, thus giving us a good reference for the stress dissipation in a material prepared by the radical cross-linking.

In the PEGDA-based hydrogels, the stress dissipation occurs mainly due to the entropic extension of pendant PEG chains around polyacrylate rods as well as due to the rod reorientation toward stress flow.^71^ In our case, however, the energy is dissipated in PEG bisdendrons-based hydrogels likely due to the physical entanglements and intrachain interactions (hydrogel bonding and van der Waals interactions),^72^ in agreement with the NMR data.

Thus, the use of bisdendrons allows us to apparently obtain step-growth polymerized materials possessing the advantages of chain-growth polymers. Indeed, the presence of clusters of reactive groups on the linear polymer extremities permits the acquisition of materials with predictable properties through better control of the stoichiometric ratio of cross-linking groups and their regular spatial organization. At the same time, the presence of long linear PEG chains brings to the material an increased pore size and high hydration degree that is beneficial for biological applications.

### Polymer Chain Dynamics in Hydrogels

To corroborate the findings and link the dynamics of the chemical structure to macromolecular rheology measurements, we investigated the polymer chain dynamics in hydrogels using NMR spectroscopy, by comparing PEG bisdendrons and 4-arm-PEG-NB-based material. To access information about the dynamics in the relevant hydrated hydrogel state, we employed magic-angle spinning (MAS) solid-state nuclear magnetic resonance. We used three types of polarization transfers to probe the polymer chain mobility inside the hydrogels: ^1^H–^13^C cross-polarization (CP), ^1^H–^13^C INEPT and ^13^C direct polarization (DP). First, the PEG signal was not observed in ^13^C CP experiments for 4-arm-PEG-NB at 5, 10 and 20% PEG concentration, as well as for 4kNBA and 4kNB at 10% PEG (7b and 8b, respectively). We hypothesize that large-amplitude chain motions in these hydrogels, typically faster than tens of microseconds, considerably reduce polarization transfer efficiency through dipolar couplings and thus the detection of the CP signal. In line with these results, we clearly observed the PEG signal in the INEPT spectra; such polarization transfer based on J-couplings allows the detection of large-amplitude motions at submicrosecond time scales.^73^ To assess and compare the relative mobility of PEG chains between the different samples, we derived the intensity ratio of INEPT/DP (Figure 4). For 4-arm-PEG-NB, we observed a comparable internal chain mobility between the material obtained at 5 and 10% PEG concentration. However, for the 4kNBA and 4kNB hydrogels, a considerably lower INEPT/DP ratio was detected. It indicates that PEG chains undergo a reduction of their fast dynamics in the PEG bisdendron material. In line with the strain measurements (γ_c_ and γ_WSO_), we interpreted these MAS solid-state NMR observations by the presence of weak interactions favored in PEG bisdendrons material, such as noncovalent interactions, possibly reducing the large-amplitude mobility of PEG chains. We note that the bisdendron material investigated here behaves differently compared to polyethylene glycol diacrylate (PEGDA) hydrogels, also studied by us using MAS NMR in López-Serrano et al.^17^ PEGDA hydrogels, although exhibiting a comparable elasticity of a few kPa, showed significantly more rigidity at the level of their PEG chain as seen by CP MAS NMR. It has been proposed that putative stiff PA rods of anisotropic nature in hydrogels might considerably influence the PEG entropy and subsequent motion restrictions by steric effects,^71^ and we hypothesize that the chemical nature of bisdendron PEG material is less prone to form such an interconnected chain network.

**Figure 4:**
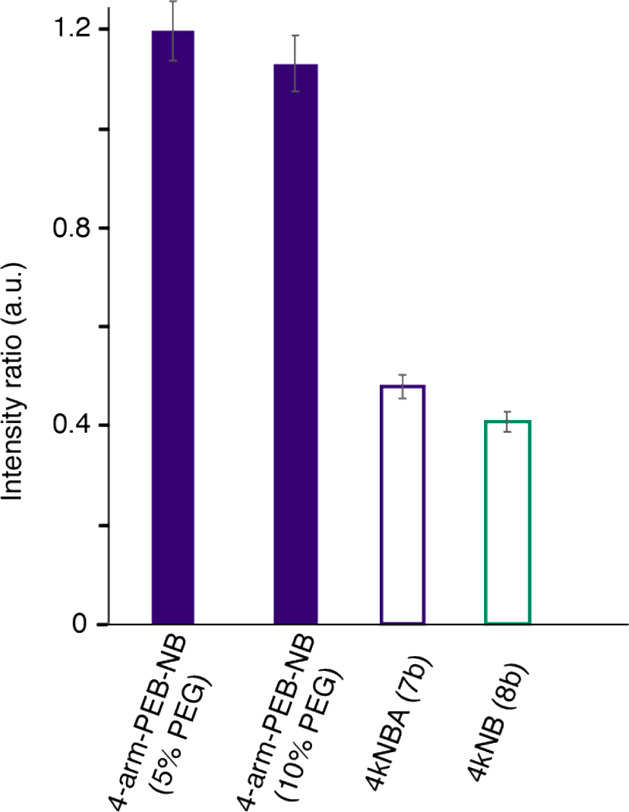
Solid-state NMR investigation of polymer chain dynamics. Intensity ratio of the PEG signal between ^13^C INEPT and direct polarization. Data recorded at 500 MHz at a MAS spinning frequency of 11 kHz at room temperature.

### Hydrogel Ultrastructure

Hydrogel porosity emerges as a fundamental design parameter that orchestrates multiple aspects of spheroid, organoid, and tumoroid behavior by directly controlling mass transport limitations that affect cell viability and growth patterns in 3D cultures. The optimal porosity depends on the specific tissue type, developmental stage, and intended application.^74^ Recent studies demonstrate that macroporous hydrogels with interconnected pore networks dramatically improve nutrient distribution and waste removal.^75,76^ Spheroids cultured in hydrogels with average pore sizes of 120 μm show enhanced paracrine effects and better cell–cell interactions compared to nanoporous systems (∼5 nm).^77^

Natural matrices like collagen and Matrigel provide inherent bioactivity but with less controllable porosity. Broguiere and co-workers found that a Matrigel culture system had a pore size smaller than 200 nm, or the resolution limit of their confocal microscope, but that a fibrin-based material had a pore size closer to 4 μm.^78^ Synthetic hydrogel systems offer precise control over porosity while overcoming polydispersity issues associated with natural matrices. Engineered PEG-based systems can be designed with specific pore sizes and degradation kinetics to optimize organoid culture.^77,79^

Freeze-fracture cryo-SEM imaging was employed to assess the internal architecture of PEG-norbornene-based hydrogels with distinct precursor topologies and molecular weights (Figure 5). All samples displayed a porous morphology. The 4-arm-PEG-NB hydrogel (Figure 5a) had relatively compact pores (1.2 ± 0.2 μm), whereas pores in hydrogels based on bisdendrons were significantly bigger (5–10 μm), thus supporting our initial hypothesis on the impact of precursor topology on the hydrogel porosity.

**Figure 5:**
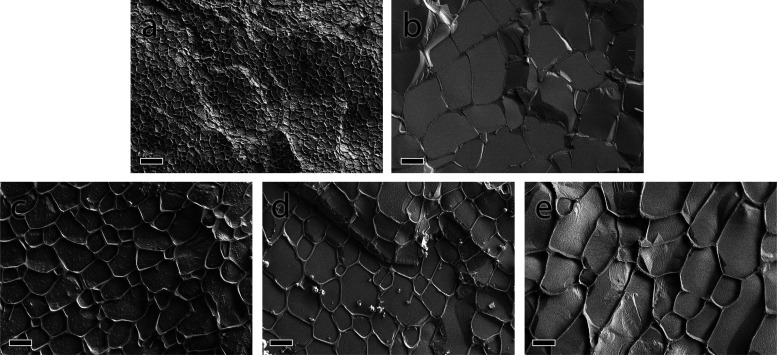
Representative freeze-fracture cryo-SEM images of hydrogels based on 4-arm-PEG-NB (a), 2kNBA (b), 4kNB (c), 4kNBA (d), and 10kNBA (e). Total PEG concentration was 10%. Scale bar = 4 μm.

Comparing pore sizes in hydrogels 4kNB (5.1 ± 1.1 μm, Figure 5c) and 4kNBA (5.4 ± 1.5 μm, Figure 5d) containing fewer or more hydrophilic surface groups, we have not found a statistically significant difference between the two values. This suggests that, despite the impact on mechanical properties (namely, the stress dissipation in the NLVR) and swelling behavior, the nature of surface groups has no effect on the hydrogel porosity.

The 10kNBA hydrogel (Figure 5e) displays a markedly different morphology, with substantially larger pores of 10.5 ± 3.2 μm. This highly open porosity matches the soft mechanical properties and enhanced swelling capacity of the long-chain bisdendron system.

Interestingly, in the 2kNBA hydrogel (Figure 5b), heterogeneous pores of 7.5 ± 1.9 μm size were observed. This value, outlying the general trend, though insignificantly different from 4kNB and 4kNBA hydrogels, is likely explained by the PEG length being insufficient to cross-link the 4-arm-PEG-SH into a dense structure.

While very small pores, such as those in Matrigel (<200 nm), result in high colony-forming efficiencies, synthetic hydrogels with pore sizes ca. 10 μm support initial cell attachment and proliferation with high seeding efficiencies, which is especially beneficial in the early stages of organoid formation. Comparative studies indicate that this pore size not only influences cell viability and differentiation, but also shapes the structural and functional maturation of organoids.^80^ This offers greater flexibility for tailoring to specific organoid models, balancing the need for strong cell–cell contacts with effective nutrient transport and tissue organization.

### Hydrogels Functionalized with a Bioactive Peptide

The use of synthetic hydrogels as matrices for cell cultivation requires the incorporation of bioactive signals to enable cellular recognition of the material by cells or to deliver specific stimuli.^77,81^ Introducing biological or biomimetic molecules into the hydrogel facilitates cell adhesion, organoid/tumoroid survival, and increases cell viability and proliferation.^15^ Concentration and spacing of these cues can be changed independently.^41^

Bioactive signals commonly used in hydrogels include adhesion factors, growth factors, or morphogenic proteins. Adhesion peptides are the most prevalent bioactive factors, including the integrin-binding RGD (Arg-Gly-Asp) motif, IKVAV, YIGSR, AG73, GFOGER, and PHSRN sequences. These peptides facilitate integrin-mediated cell adhesion and mechanotransduction.^19,82–88^ Growth factors such as VEGF, EGF, FGF, PDGF, HGF, and IGF are incorporated to stimulate proliferation, differentiation, and tissue-specific functions^77,81,89^ The use of short peptides instead of full-length proteins permits the introduction of more bioactive signals into a hydrogel and to avoid nonspecific signaling.

From a mechanistic perspective, two primary factors influence the cellular response to peptide-modified hydrogels, aside from the peptide sequence itself: the overall peptide concentration within the hydrogel and the local peptide distribution. The latter refers to whether peptide molecules are dispersed individually or are organized into clusters or islets. Given that integrins are usually clustered in the cell membrane, hydrogels bearing small peptide clusters provide a better response of seeded cells.^30^ While this concept has been explored in some neural and tissue engineering models,^90^ it has not yet been applied to organoid, tumoroid, or spheroid hydrogel systems as of the present date. Recent reviews continue to highlight the lack of cluster formation and multifunctionalization in the state-of-the-art, pointing to an important gap in the field.^81^

Herein, we selected the most commonly used peptide for organoid culture, the RGD peptide, for immobilization. Specifically, we focused on a cell adhesion peptide containing the RGD motif, with the sequence GRGDSPC, to be incorporated into the hydrogels. Our design introduces a cysteine at the C-terminus to provide a convenient way to graft the PEG-norbornene precursors through the same thiol–ene chemistry approach as that used for the hydrogel formation. The UV-assisted grafting of the peptide has been done prior to the cross-linking (Figure 1). This permitted us to assess the efficiency of peptide grafting by HPLC (Figure 6 and Figure S6).

**Figure 6:**
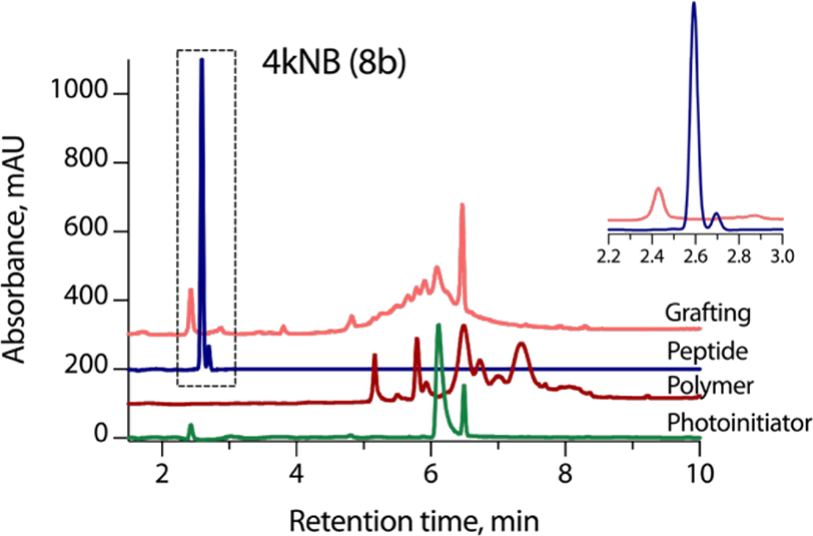
HPLC profiles (λ = 214 nm) of the PEG-norbornene precursor 4kNB, GRGDSPC peptide, photoinitiator (LAP) and peptide-PEG-conjugate. Selected zone is zoomed in in the inset. Gradient: 5–100% CH_3_CN in H_2_O with 0.1% TFA, 10 min.

The comparison of HPLC profiles revealed the complete consumption of the peptide upon grafting. This result is valuable as it proves the absence of free peptide in hydrogel samples. Free adhesion peptide is known to saturate the integrins on the cell surface, thus preventing cells from adhering to the material.

The data on the peptide grafting efficiency permitted us to estimate the global peptide concentration in a hydrogel, taking into account the increase in the material volume upon swelling (Table 1). The global peptide concentration was 1.5 mM in as-prepared hydrogels and 0.28–0.8 mM in abundantly swollen ones. Importantly, the peptide grafting does not drastically change the mechanical properties of the material (Figure S7).

The peptide concentration values given above may seem lower than those reported elsewhere for organoid maintenance (for instance, 2 mM for the blend RGDS, IKVAV, and GFOGER in ref 22). However, in ref 22, the authors give the nominal peptide concentration before swelling, without taking into account the concentration decrease due to the volume stretch. The apparent peptide concentration in their case might be closer to that we report herein.

The temporal stability of the ligand presentation remains to be established. Previous work on PEG hydrogels indicates that covalently bound adhesive peptides can be retained over days once the initial unbound fraction has diffused out, yet cell behavior is highly sensitive to the persistence or removal of these cues.^91^

To prove our hypothesis concerning the impact of norbornene dendritic units on the peptide clusterization, we have compared the distribution of TAMRA-labeled RGD peptide in hydrogels based on 4-arm-PEG-NB and 10kNBA precursors using confocal fluorescence microscopy (Figure 7). The confocal microscopy images revealed high-intensity multipixel spots, which we assigned to peptide clusters, in two materials. The occurrence of these spots was significantly higher in 10kNBA gels (100–650 units per nanoliter) than in 4-arm-PEG-NB gels, which is consistent with the presence of norbornene clusters at the ends of long linear PEG chains.

**Figure 7:**
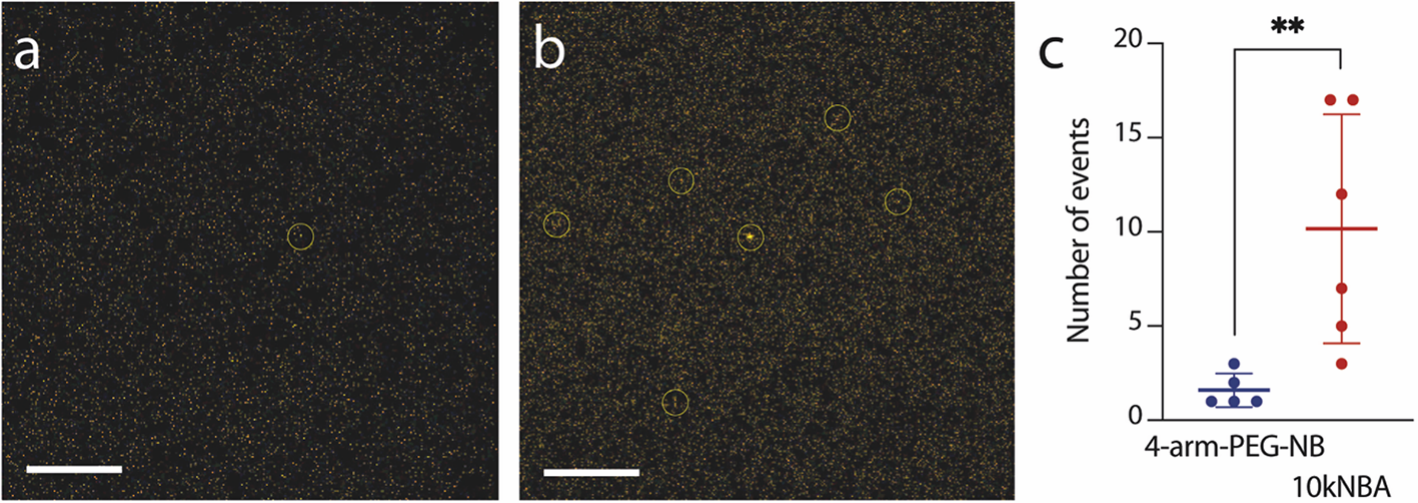
Representative confocal microscopy images of hydrogels based on 4-arm-PEG-NB (a) and 10kNBA (b) containing TAMRA-labeled RGD peptide. Total PEG concentration 10%. Scale bar represents 5 μm. Circle depicts high-intensity multipixel spot assigned to a peptide cluster. (c) Comparison of the peptide cluster occurrence in observed hydrogels, **p < 0.01. See the Experimental Section and the Supporting Information for more details.

In combination with cryo-SEM data showing larger pores in 10kNBA hydrogels and rheological measurements evidencing abundant weak interactions and enhanced chain mobility, these observations support the idea that dendritic norbornene units promote local enrichment and clusterization of active principles (adhesion peptides, growth factors, and tissue-specific biochemical signals) rather than their uniform dispersion. Such a clustered presentation is expected to favor multivalent integrin engagement and mechanotransduction compared to a topologically similar matrix with the same global peptide concentration but a more homogeneous ligand distribution and therefore provides a rational handle to decouple overall peptide content from its local organization in future organoid and tumoroid culture studies.

## Conclusions

This study presents a versatile hydrogel platform built from norbornene-functionalized PEG bisdendrons, designed to tackle the central limitations of conventional PEG-based matrices in 3D cell culture applications. By manipulating PEG chain length and molecular topology, norbornene-containing PEG bisdendrons appeared to yield hydrogels with modulable elasticity (30 Pa to 3.3 kPa) and unique capability for dissipating external stress that can be achieved by the rational choice of the PEG precursor length and functionalization type. The properties of bisdendron-based hydrogels differ notably from those of hydrogels based on branched PEG-norbornene currently in use for cell culture. For instance, critical strain positioning and pore diameter for 10kNBA were 113% and 10.5 μm vs 21.5% and 1.2 μm for 4-arm-PEG-NB.

The viscoelastic behavior of these hydrogels, characterized by variable relaxation dynamics (τ_1/2_ = 20–673 s), provides a mechanical response that is critical for cell viability and organoid maturation and development.

The bisdendron architecture provides a versatile platform for the incorporation of diverse bioactive peptides or peptide cocktails, enabling customization of the hydrogel microenvironment for specific applications. Importantly, due to the presence of norbornene clusters, peptide islands can be formed in the hydrogel volume. This feature may promote ligand binding and cell survival. The modular design enables on-demand incorporation of ECM-mimicking signals, permitting the potential use of hydrogels in organoid mechanobiology and phenotyping studies, as well as cell–matrix interactions assays.

Altogether, PEG bisdendron hydrogels stand as prospective synthetic analogs of ECM, offering structural versatility, tunable biophysics, and biochemical modularity. In the future, these materials can give the opportunity to create matrix libraries with tunable mechanical, degradative, and bioactive profiles, ultimately enhancing the physiological relevance of in vitro tissue models and enabling precise studies of tissue development, disease progression, and drug responses.

## Experimental Section

### General Considerations

All manipulations were carried out using a standard dry nitrogen-high vacuum technique. Organic solvents were dried and freshly distilled under nitrogen prior to use. Reagents and PEG precursors (MW 2000, 4000, and 10,000) were obtained from commercial sources (Sigma-Aldrich or TCI, purity > 98%) and used as received. 4-arm-PEG derivatives containing norbornene residues (4-arm-PEG-NB, MW 10,000) and thiol groups (4-arm-PEG-SH, MW 20000) were purchased from Biopharma PEG (US) and handled according to the manufacturer’s instructions. GRGDSPC and K­(TAMRA)­GRGDSPC peptides (98%) were obtained from GeneCust (France) and handled according to the manufacturer’s instructions.

### Analytical Techniques

#### NMR

^1^H solution NMR spectra were recorded on a Bruker AV300 SB spectrometer (Bruker, Karlsruhe, Germany). ^1^H chemical shifts (δ, ppm) were measured relative to the residual resonances of solvents. ^13^C solid-state NMR spectra were recorded at 500 MHz ^1^H frequency on a Bruker spectrometer, using 11 kHz spinning frequency, 30 ms acquisition, and 10k scans. CP was achieved with a 1 ms contact time.

#### Raman Spectroscopy

The Raman analyses were performed using a WITec Alpha 300RS (WITec GmbH, Ulm, Germany) instrument equipped with an EMCCD. Raman spectra were recorded from 200 to 3700 cm^–1^ (600 g per mm grating, spectral resolution of 8 cm^–1^) by using a 532 nm excitation wavelength. The beam was focused on the sample with a Zeiss objective (20×/0.4 NA), and the laser intensity at the sample was approximately 30 mW. Several spectra were acquired with a 1 to 10 s acquisition time per spectrum to check for signal heterogeneity at different localizations on the sample and to ensure that no laser-induced change occurred on the spectra. All data were processed for background subtraction (using a shape function) using WITec Project FIVE Plus software (WITec GmbH, Ulm, Germany).

#### HPLC

Analytical RP-HPLC analyses were performed on a Dionex U3000SD instrument using a Macherey-Nagel Nucleodur column (4.6 × 100 mm, 3 μm) at a flow rate of 1 mL/min. The mobile phase was composed of 0.1% (v/v) trifluoroacetic acid (TFA) in Milli-Q water and 0.1% TFA in CH_3_CN.

#### Cryo-SEM

To perform scanning cryo-electron microscopy, samples were mounted with a mixture of tissue freezing medium and colloidal graphite on a specific shuttle, cryofixed by slush freezing, and then fractured with a scalpel. The shuttle was transferred into the preparation chamber (−140 °C) and sublimated for 6.5 min at −95 °C. Samples were then transferred to the cryostage (CRYO-SEM PP3010T, Quorum Technologies, England). Observations were done at 0.8 kV with a GeminiSEM 300_FESEM instrument (ZEISS, Germany). To assess quantitative data on the pore size, hydrogel pores were measured using ImageJ software (at least 30 measurements, 3 observation zones).

#### Confocal Microscopy

For confocal imaging, hydrogel samples containing TAMRA-labeled peptide were observed on a ZEISS LSM 800 Airyscan inverted laser-scanning confocal microscope equipped with a Plan-Apochromat 40×/1.4 oil immersion objective (working distance 130 μm, oil immersion medium, refractive index 1.518). Image stacks (512 × 512 pixels, 16-bit depth, single channel) were acquired with an optical zoom of 3.4, corresponding to a lateral pixel size of 0.098 μm in the *X*–*Y* plane and a *Z* step of 0.26 μm over 50 optical sections. TAMRA was excited at 543 nm, and emission was collected at 567 nm under epifluorescence illumination, using a pinhole size of 35.8 μm and Airyscan detection mode. Confocal image stacks were analyzed using an automated outlier-detection workflow (see the Supporting Information) to quantify the formation of peptide clusters within hydrogels. First, image stacks were converted into intensity histograms to calculate the global mean fluorescence and standard deviation for each data set. Voxels whose intensity exceeded the mean + 3 × SD threshold were identified, and spatially connected voxels above this threshold were grouped into 3D objects; only components with a minimum volume of more than four pixels were retained and assigned as peptide clusters. For each hydrogel formulation, the number of such high-intensity clusters was counted per sample (*n* = 5–6) and used as the readout of peptide island occurrence. The results of the automated images treatment in studied samples have been published in an open repository. Statistical comparison between conditions was performed by using the Mann–Whitney criterion to assess differences in cluster abundance.

### Synthetic Procedures

#### 6-(5-Norbornene-2,3-dicarboximido)­hexanoic Acid (**2**)

5-Norbornene-2,3-dicarboxylic anhydride (1) (3.28 g, 20 mmol) and 6-aminohexanoic acid (2.62 g, 20 mmol) were mixed in 45 mL of acetic acid and refluxed overnight at 110 °C. The reaction was monitored by TLC (10% methanol in CH_2_Cl_2_, product Rf ≈ 0.85). After all reactants were consumed, the reaction mixture was cooled down to r.t., diluted with 300 mL of cold water, and the product was extracted with 150 mL CH_2_Cl_2_, and then the water phase was further washed twice with 50 mL of CH_2_Cl_2_. The combined organic fraction was washed with 50 mL of 1 M HCl, then twice with 100 mL of water, dried over Na_2_SO_4_, and concentrated to a yellow oil that further solidified into an off-white solid, which was washed with pentane, gathered on a glass porous filter, and dried under high vacuum. White solid, yield: 4.7 g (85%). ^1^H NMR (300 MHz, CDCl_3_) δ, ppm: 1.31 (m, 2H), 1.46 (m, 2H), 1.51–1.80 (m, 6H), 2.35 (t, *J* = 7.3 Hz, 2H), 3.21–3.30 (m, 2H), 3.35 (t, *J* = 7.3 Hz, 2H), 3.41 (s, 2H), 6.11 (d, *J* = 2.0 Hz, 2H).

#### *O*,*O*′-Bis­(toluenesulfonyl)­polyethylene Glycol (**4a**–**c**)

1.5 mmol PEG (3a-c) was dissolved in 150 mL of CH_2_Cl_2_ and cooled to 0 °C with an ice bath, then toluenesulfonyl chloride (1.43 g, 7.5 mmol) was added. The solution of triethylamine (760 mg, 1.05 mL, 7.5 mmol) in 25 mL of CH_2_Cl_2_ was added dropwise over 15 min, and the reaction mixture was left stirring for 30 min at 0 °C and then overnight at r.t. The reaction was monitored by ^1^H NMR, followed by the integration of signals corresponding to terminal ethylene glycol units and the PEG scaffold. When the reaction was considered completed, 100 mL of water was added to the reaction mixture and stirred for 15 min, followed by phase separation. The water phase was washed 3 times with 50 mL of CH_2_Cl_2_. The combined organic phase was washed consecutively with 100 mL of water, twice with 100 mL of 5% K_2_CO_3_, with 100 mL of 1 M HCl, and with 100 mL of water, dried over Na_2_SO_4_, and concentrated to an off-white solid. The solid was dispersed in 200 mL of diethyl ether upon stirring, filtered, and washed twice with 150 mL of diethyl ether and then dried under high vacuum.

##### PEG-2000 (**4a**)

White solid, yield: 87%. ^1^H NMR (300 MHz, CDCl_3_) δ, ppm: 2.49 (s, 6H), 3.68 (m, PEG), 4.20 (m, 4H), 7.39 (d, *J* = 7.8 Hz, 2H), 7.84 (d, *J* = 8.0 Hz, 4H).

##### PEG-4000 (**4b**)

White solid, yield: 90%. ^1^H NMR (300 MHz, CDCl_3_) δ, ppm: 2.49 (s, 6H), 3.68 (m, PEG), 4.16 (m, 4H), 7.35 (d, *J* = 7.8 Hz, 2H), 7.80 (d, *J* = 8.0 Hz, 4H).

##### PEG-10,000 (**4c**)

White solid, yield: 94%. ^1^H NMR (300 MHz, CDCl_3_) δ, ppm: 2.49 (s, 6H), 3.68 (m, PEG), 4.18 (m, 4H), 7.36 (d, *J* = 7.8 Hz, 2H), 7.81 (d, *J* = 8.0 Hz, 4H).

#### *O*,*O*′-Bis­(5-oxidimethylisophthalate)­polyethylene Glycol (**5a**–**c**)

PEG derivative (4a-c, 0.5 mmol) and dimethyl 5-hydroxyisophthalate (315 mg, 1.5 mmol) were dissolved in 60 mL of CH_3_CN, then K_2_CO_3_ (210 mg, 1.5 mmol) was added, and the reaction mixture was refluxed overnight at 85 °C. The reaction was monitored by the shift of signals corresponding to terminal ethylene glycol units in the ^1^H NMR spectra. Once the reaction is complete, the solvent was removed *in vacuo*, and the residue was redissolved in 150 and 100 mL of water. Once the phases are separated, the water phase was washed 3 times with 50 mL of CH_2_Cl_2_. The combined organic phase was washed twice with 100 mL of 1 M NaOH, and with 100 mL of water, dried over Na_2_SO_4_, and concentrated to an off-white solid. The solid was dispersed in 100 mL of diethyl ether upon stirring, filtered, washed twice with 100 mL of diethyl ether, and then dried under high vacuum.

##### PEG-2000 (**5a**)

White solid, yield: 65%. ^1^H NMR (300 MHz, CDCl_3_) δ, ppm: 3.68 (m, PEG), 3.93 (s, 6H), 4.20 (m, 4H), 7.76 (d, *J* = 1.5 Hz, 4H), 8.27 (t, *J* = 1.5 Hz, 2H).

##### PEG-4000 (**5b**)

White solid, yield: 70%. ^1^H NMR (300 MHz, CDCl_3_) δ, ppm: 3.63 (m, PEG), 3.92 (s, 6H), 4.20 (m, 4H), 7.76 (d, *J* = 1.5 Hz, 4H), 8.26 (t, *J* = 1.5 Hz, 2H).

##### PEG-10,000 (**5c**)

White solid, yield: 96%. ^1^H NMR (300 MHz, CDCl_3_) δ, ppm: 3.65 (m, PEG), 3.94 (s, 6H), 4.22 (m, 4H), 7.78 (d, *J* = 1.5 Hz, 4H), 8.29 (t, *J* = 1.5 Hz, 2H).

#### *O*,*O*′-Bis­(5-oxy-1,3-di­(hydroxymethyl)­phenyl)­polyethylene Glycol (**6a**–**c**)

PEG derivative (*5a*, 0.5 mmol; *5b*, 0.44 mmol; *5c*, 0.3 mmol) was dissolved in 100 mL anhydrous THF under N_2_, then borane-dimethylsulfide complex (11 equiv) was added dropwise, and the reaction mixture was refluxed overnight at 80 °C. The reaction was monitored following the signals of methyl ester protons and benzyl alcohol protons in the ^1^H NMR spectra. Once the reduction is complete, the reaction mixture was cooled down to r.t., and then to 0 °C with an ice bath; then, 30 mL absolute CH_3_OH was added dropwise and left for 2 h upon vigorous stirring. The volatiles were removed *in vacuo*, and the viscous oily residue was dispersed in 150 mL of diethyl ether upon stirring, filtered, washed twice with 100 mL of diethyl ether, and then dried under high vacuum.

##### PEG-2000 (**6a**)

White solid, yield: 98%. ^1^H NMR (300 MHz, CDCl_3_) δ, ppm: 3.67 (m, PEG), 4.18 (t, 4H), 4.67 (d, *J* = 4.1 Hz, 8H), 6.90 (s, 4H), 6.98 (s, 2H).

##### PEG-4000 (**6b**)

White solid, yield: 98%. ^1^H NMR (300 MHz, CDCl_3_) δ, ppm: 3.65 (m, PEG), 4.15 (s, 4H), 4.63 (d, *J* = 4.1 Hz, 8H), 6.86 (s, 4H), 6.95 (s, 2H).

##### PEG-10,000 (**6c**)

White solid, yield: 98%. ^1^H NMR (300 MHz, CDCl_3_) δ, ppm: 3.65 (m, PEG), 4.16 (m, 4H), 4.67 (d, *J* = 4.1 Hz, 8H), 6.86 (s, 4H), 6.96 (s, 2H).

#### Carboxy-norbornene Polyethylene Glycol Tetraester (**7a**–**c**)

PEG derivative (**5a**–**c**, 0.14 mmol) was dissolved in 40 mL of CH_2_Cl_2_ and cooled to 0 °C with an ice bath, and then 5-norbornene-2,3-dicarboxylic anhydride (**1**) (185 mg, 1.12 mmol) was added. The solution of triethylamine (226 mg, 0.32 mL, 2.24 mmol) in 10 mL CH_2_Cl_2_ was added dropwise over 15 min, and the reaction mixture was left stirring for 30 min at 0 °C and then 2 days at r.t. The reaction was monitored following the signals of benzyl alcohol protons in the ^1^H NMR spectra. When the reaction was considered completed, 40 mL of water was added to the reaction mixture and stirred for 30 min, followed by phase separation. The water phase was washed 3 times with 30 mL of CH_2_Cl_2_. The combined organic phase was washed twice with 50 mL of 1 M HCl, dried over Na_2_SO_4_, and concentrated to a clear oil that further solidified into an off-white solid. The solid was dispersed in 120 mL of diethyl ether upon stirring, filtered, washed twice with 100 mL of diethyl ether, and then dried under high vacuum.

##### PEG-2000 (2kNBA, **7a**)

White solid, yield: 76%. ^1^H NMR (300 MHz, CDCl_3_) δ, ppm: 1.35 (m, 4H), 1.48 (m, 4H), 1.56 (m, 8H), 3.19 (m, 8H), 3.33 (m, 8H), 3.64 (m, PEG), 4.12 (t, 4H), 4.79 (m, 4H), 5.00 (m, 4H), 6.03–6.37 (m, 8H), 6.79 (m, 6H).

##### PEG-4000 (4kNBA, **7b**)

White solid, yield: 79%. ^1^H NMR (300 MHz, CDCl_3_) δ, ppm: 1.35 (m, 4H), 1.48 (m, 4H), 3.15 (m, 8H), 3.33 (m, 8H), 3.66 (m, PEG), 4.33 (t, 4H), 4.79 (m, 4H), 5.00 (m, 4H), 6.03–6.37 (m, 8H), 6.79 (m, 6H).

##### PEG-10,000 (10kNBA, **7c**)

White solid, yield: 96%. ^1^H NMR (300 MHz, CDCl_3_) δ, ppm: 1.35 (m, 4H), 1.48 (m, 4H), 3.14 (m, 8H), 3.32 (m, 8H), 3.66 (m, PEG), 4.32 (t, 4H), 4.80 (m, 4H), 5.00 (m, 4H), 6.03–6.40 (m, 8H), 6.79 (m, 6H).

#### Norbornene Polyethylene Glycol Tetraester (**8a**–**c**)

6-(5-Norbornene-2,3-dicarboximido)­hexanoic acid (2, 366 mg, 1.32 mmol) was dissolved in 40 mL dry CH_2_Cl_2_ under N_2_ and cooled to 0 °C with ice bath, then oxalyl chloride (420 mg, 0.3 mL, 3.3 mmol) was added, and the reaction mixture was left stirring 30 min at 0 °C, then overnight at r.t. When the ^1^H NMR spectra showed full conversion of the acid into the corresponding chloroanhydride, the volatiles were removed.

The residue obtained was dissolved in 40 mL dry CH_2_Cl_2_ under N_2_ and cooled to 0 °C with an ice bath. Then, the mixture of PEG derivative (**6a**–**c**, 0.22 mmol) and triethylamine (222 mg, 0.31 mL, 2.2 mmol) in 40 mL CH_2_Cl_2_ was added dropwise over 1 h, and the reaction mixture was left stirring 2 h at 0 °C and then overnight at r.t. The reaction was monitored following the signals of benzyl alcohol protons in the ^1^H NMR spectra. When the reaction was considered completed, 100 mL of water was added to the reaction mixture and stirred for 30 min, followed by phase separation. The water phase was washed 3 times with 50 mL of CH_2_Cl_2_. The combined organic phase was washed twice with 100 mL of 5% K_2_CO_3_, 100 mL of 1 M HCl, and 100 mL of water, dried over Na_2_SO_4_, and concentrated *in vacuo*. The residue was dispersed in 120 mL of diethyl ether upon stirring, filtered, washed twice with 100 mL of diethyl ether, and then dried under high vacuum.

##### PEG-2000 (2kNB, **8a**)

White solid, yield: 95%. ^1^H NMR (300 MHz, CDCl_3_) δ, ppm: 1.32 (m, 4H), 1.47 (m, 4H), 1.51–1.80 (m, 12H), 2.36 (m, 4H), 2.45 (m, 4H), 3.26 (m, 4H), 3.34 (m, 4H), 3.40 (s, 4H), 3.67 (m, PEG), 4.15 (m, 4H), 5.07 (s, 8H), 6.11 (s, 4H), 6.87 (s, 4H), 6.92 (s, 2H).

##### PEG-4000 (4kNB, **8b**)

White solid, yield: 95%. ^1^H NMR (300 MHz, CDCl_3_) δ, ppm: 1.32 (m, 4H), 1.46 (m, 4H), 1.51–1.80 (m, 12H), 2.32 (m, 4H), 2.45 (m, 4H), 3.25 (m, 4 Hz), 3.34 (m, 4H), 3.40 (s, 4H), 3.66 (m, PEG), 4.21 (m, 4H), 5.07 (s, 8H), 6.11 (s, 4H), 6.87 (s, 4H), 6.91 (s, 2H).

##### PEG-10,000 (10kNB, **8c**)

White solid, yield: 96%. ^1^H NMR (300 MHz, CDCl_3_) δ, ppm: 1.32 (m, 4H), 1.46 (m, 4H), 1.51–1.80 (m, 12H), 2.44 (m, 4H), 3.26 (m, 4 Hz), 3.34 (m, 4H), 3.40 (s, 4H), 3.66 (m, PEG), 4.23 (m, 4H), 5.07 (s, 8H), 6.11 (s, 4H), 6.87 (s, 4H), 6.95 (s, 2H).

#### Hydrogel Preparation

PEG-norbornene polymers (4-arm-PEG-NB, 7a-c, 8a-c) and 4-arm-PEG-SH were dissolved in 1x phosphate-buffered saline (PBS, 10 mM Na-phosphate, 13.7 mM NaCl, and 2.9 mM KCl) containing 0.1% lithium phenyl-2,4,6-trimethylbenzoylphosphinate (LAP) as a photoinitiator. The quantities of components were optimized to obtain the total PEG concentration of 5 or 10%, keeping the norbornene:thiol ratio equimolar. The precursor solutions were cast into the wells of silicon molds (Ø 8 mm, height 1.8 mm) and irradiated with 365 nm UV light (4.5 mW/cm^2^) for 4 min. The materials formed were abundantly swollen in PBS prior to further manipulations. Norbornene-containing PEG bisdendrons from different batches were used for hydrogel formation.

To obtain peptide-containing hydrogels, PEG-norbornene polymers were first mixed with the GRGDSPC peptide or K­(TAMRA)­GRGDSPC peptide in PBS containing 0.1% LAP, and the solution was irradiated with 365 nm UV light (4.5 mW/cm^2^) for 4 min and then mixed with 4-arm-PEG-SH, and the hydrogel materials were prepared as described above. To determine the completeness of peptide grafting, peptide-conjugated PEG-norbornene samples were analyzed by HPLC.

#### Ellman’s Test

Freshly prepared hydrogels were placed into microplate wells and soaked in 0.1 M sodium phosphate, pH 8.0, containing 1 mM EDTA, and Ellman’s reagent (Thermo Scientific) solution was added according to the manufacturer’s protocol. As a standard, the same test with corresponding quantities of 4-arm-PEG-SH was done. The samples were incubated for 30 min, and then optical absorbance at 412 nm was measured using a Synergy HTX microplate reader. The sample/standard ratio of absorbance values was used to quantify the amount of free thiol in the hydrogels. All experiments were run in tetraplicates.

#### Rheological Measurements

Rheological measurements were carried out on a Discovery hybrid rheometer HR-1 (TA Instruments) using 8 mm parallel cross-hatched geometry. To fit the geometry, swollen hydrogel materials were cut with a Ø 8 mm hole punch.

The data were acquired at 20 °C, with the temperature being controlled by a Peltier plate.

First, an amplitude sweep (0–20% strain) was performed to determine a linear viscoelastic region. Then, within the linear viscoelastic region, oscillatory frequency sweeps were then carried out under a constant strain of 3% in the angular frequency range of 0.1–100 rad/s.

Stress-relaxation experiments were performed by inducing a shear strain of 3%.

High-strain amplitude sweeps (up to 1000%) were performed at an angular frequency of 10 rad/s.

## Supplementary Material



## Notes

Raman spectra, Ellman’s test results, swelling profiles, rheological data, HPLC profiles, NMR spectra, and confocal microscopy data. The automated image treatment algorithm can be accessed by the following link: https://gitlab.xlim.fr/asali_rgd/rgd_peptide_clustering_confocal (PDF)
