# Supplementary material for: Adaptive PEG Bis-dendron Hydrogels with Tunable Mechanics and Bioactivity
Source: Chem Mater. 2026 Apr 15;38(8):4319–33. doi: 10.1021/acs.chemmater.6c00419 (PMC13131979; doi:10.1021/acs.chemmater.6c00419)
Supplement: Supplementary file 1 [file cm6c00419_si_001.pdf]

Adaptive PEG Bis-Dendron Hydrogels with Tunable Mechanics and Bioactivity

Evgeny Apartsin[1,*], Noël Richard[2], Birgit Habenstein[3], Antoine Loquet[3,4],
Sophie Lecomte[1], Marie-Christine Durrieu[1,*]

[1]Univ. Bordeaux, CNRS, Bordeaux INP, CBMN, UMR 5248, F-33600 Pessac, France
[2]Univ. Poitiers, CNRS, XLIM, UMR 7252, F-86360, Futuroscope Chasseneuil, France
[3]Univ. Bordeaux, CNRS, Bordeaux INP, CBMN, UMR 5248, IECB, F-33600 Pessac, France
[4]Univ. Bordeaux, CNRS, INSERM, IECB, US1, UAR 3033, F-33600 Pessac, France

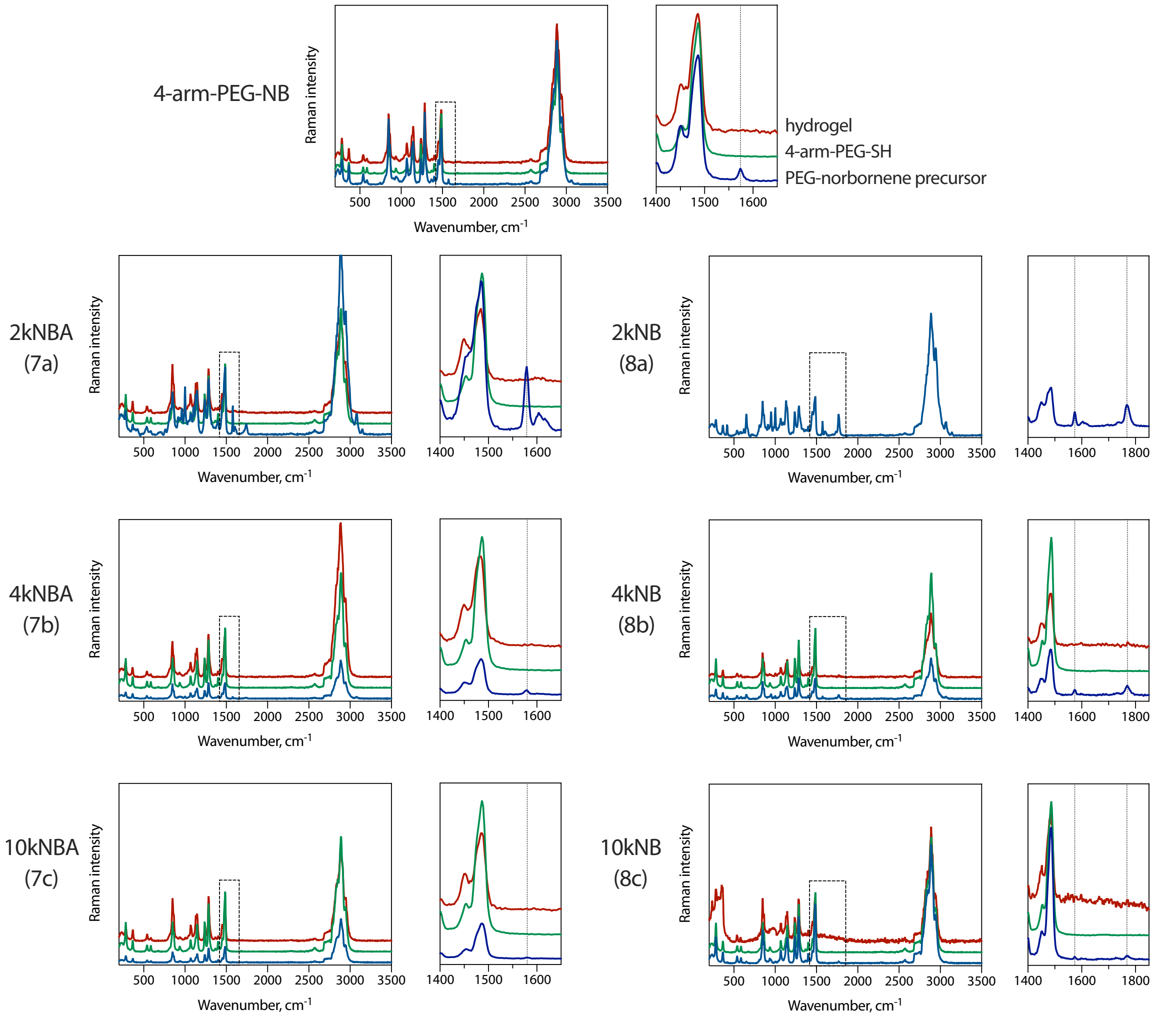

Figure S1. Raman spectra of the PEG-norbornene precursors, PEG-thiol precursor 4-arm-PEG-SH and their hydrogels. Selected zones (dashed rectangles) are zoomed.

Table S1. Residual thiol content after the gelation, as quantified by the Ellman's test.

PEG-norbornene precursor	Resudual thiols, %
4-arm-PEG-NB	1.7 ± 0.3
2kNBA (7a)	1.2 ± 0.1
4kNBA (7b)	0.8 ± 0.2
10kNBA (7c)	0.9 ± 0.2
2kNB (8a)[a]	
4kNB (8b)	1.3 ± 0.3
10kNB (8c)	0.7 ± 0.2

[a] Not assessed due to the incomplete gelation

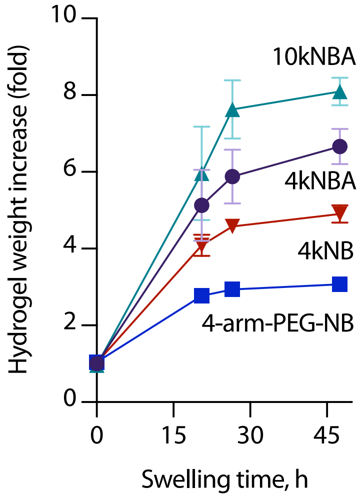

Figure S2. Representative swelling profiles of as-prepared hydrogels in PBS. Data presented as mean±s.d. (n=4).

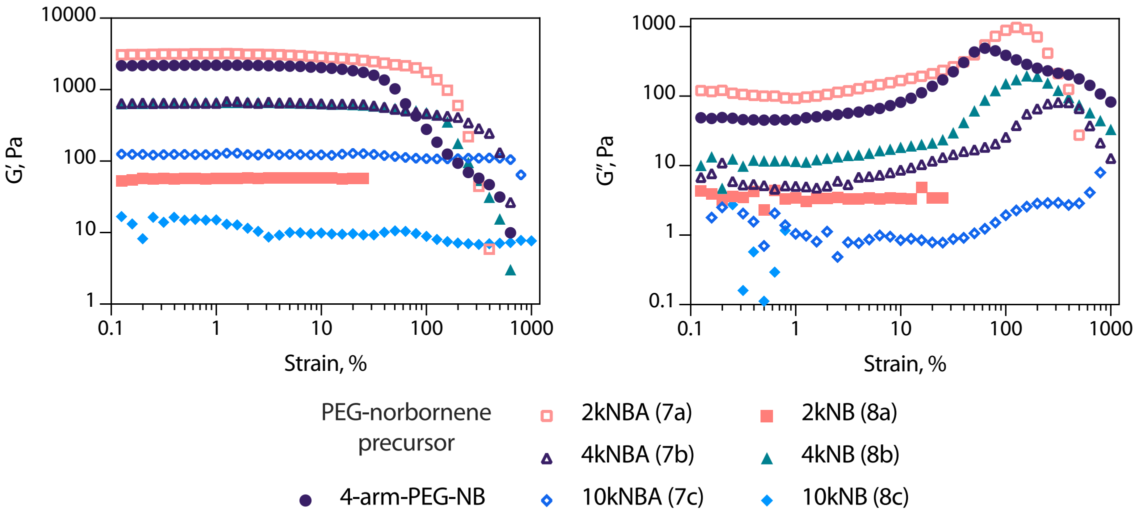

Figure S3. Strain sweep rheology profiles for hydrogels prepared from PEG-norbornene and 4-arm-PEG-SH precursors. Oscillation frequency: 1 Hz.

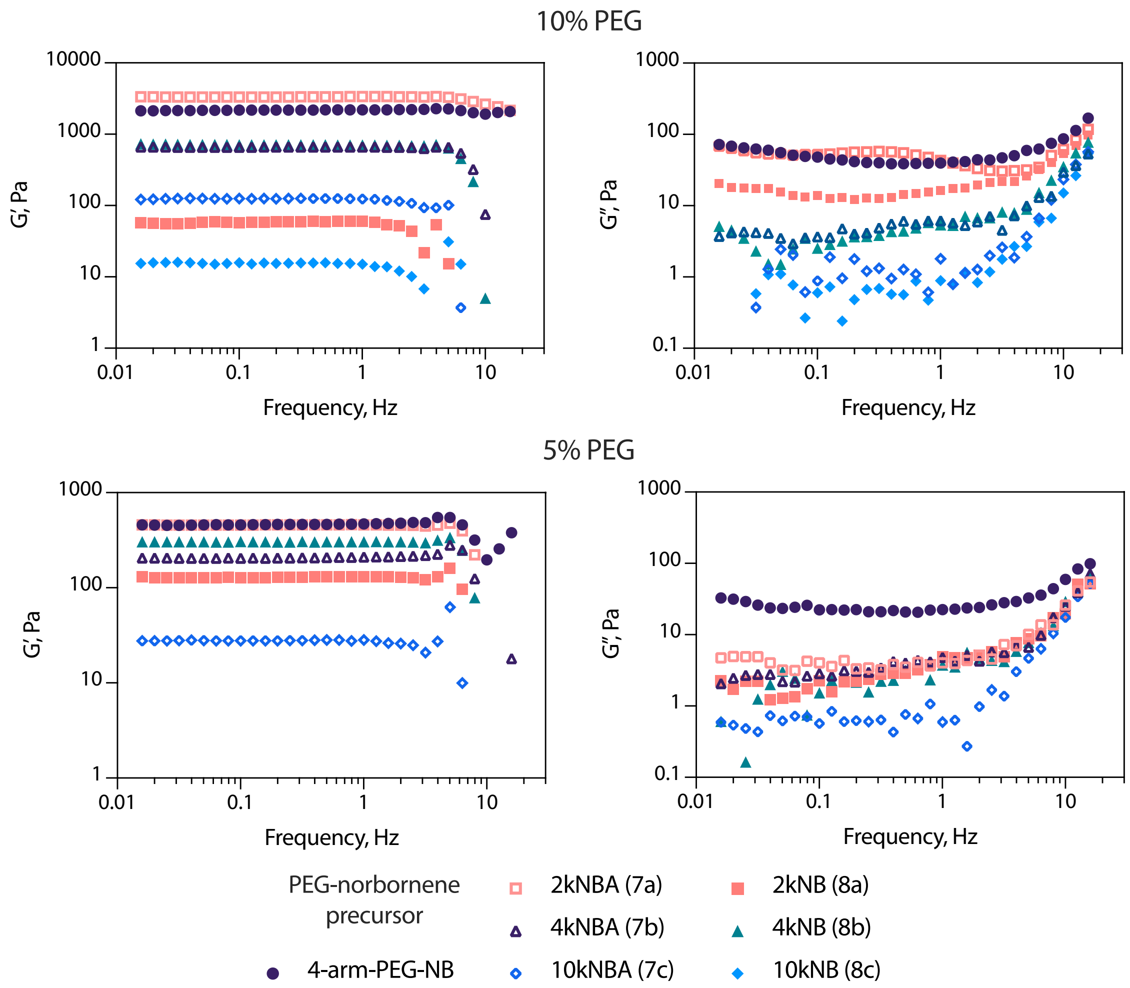

Figure S4. Oscillatory shear rheology profiles for hydrogels prepared from PEG-norbornene and 4-arm-PEG-SH precursors. Oscillation strain: 3%.

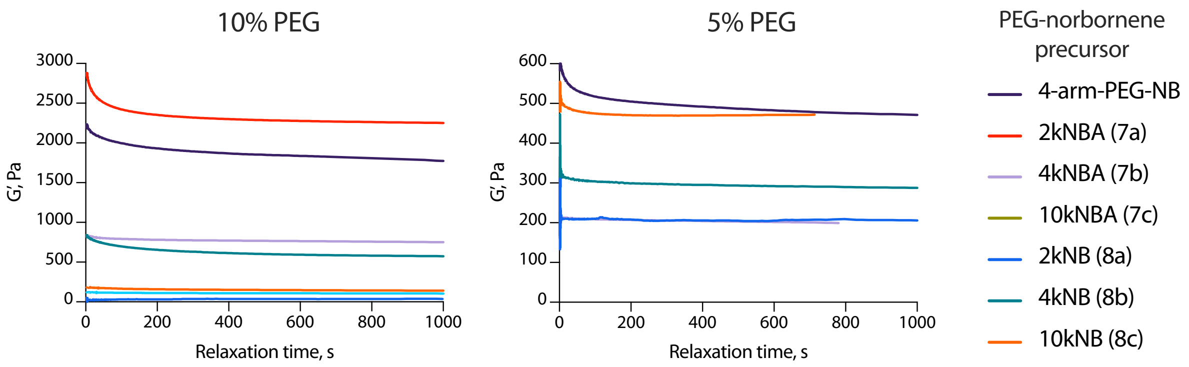

Figure S5. Stress relaxation profiles in PEG-norbornene-based hydrogels. Shear strain: 3%.

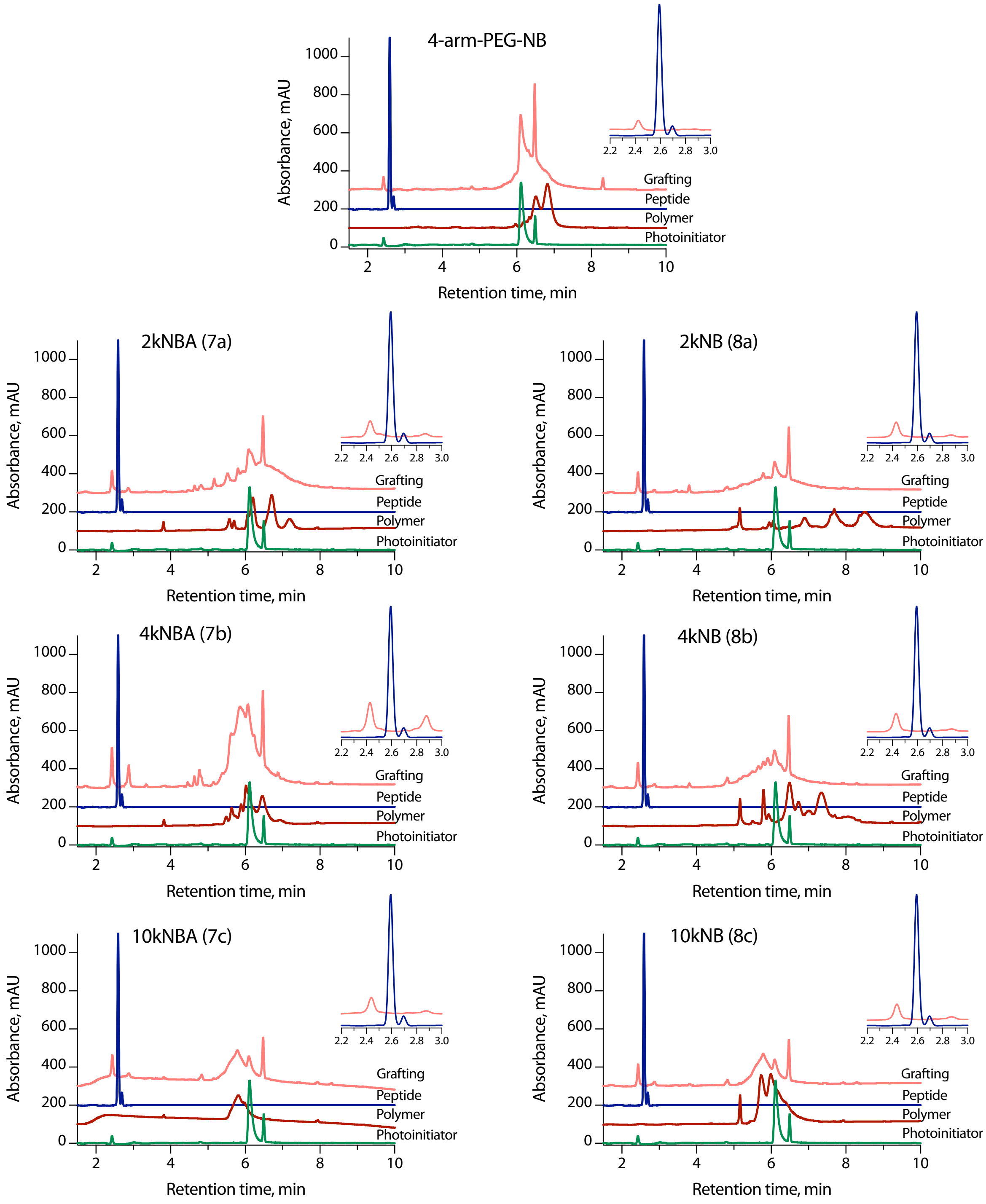

Figure S6. HPLC profiles (λ = 214 nm) of PEG-norbornene precursors, GRGDSPC peptide, photoinitiator (LAP) and peptide-PEG-conjugate. Selected zone is zoomed in the inset. Gradient: 5-100% MeCN in H_2O with 0.1% TFA, 10 min.

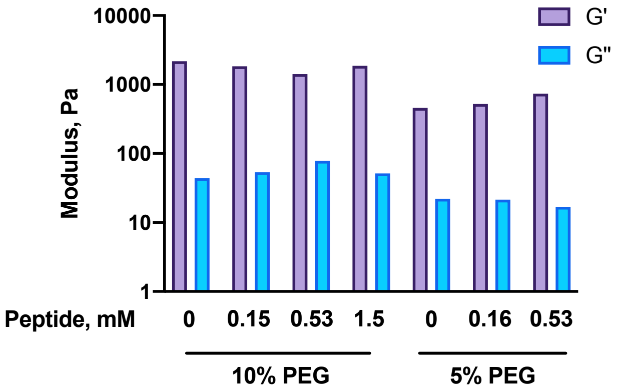

Figure S7. Mechanical properties of 4-arm-PEG-NB-based hydrogels functionalized with the adhesion peptide. Oscillatory shear rheology, strain: 3%, frequency: 1Hz.

Treatment of the confocal microscopy images

Confocal image stacks were analyzed using an automated outlier-detection workflow (see below) to quantify peptide cluster formation within hydrogels. First, image stacks were converted into intensity histograms to calculate the global mean fluorescence and standard deviation for each dataset. Voxels whose intensity exceeded the Mean+3×S.D. threshold were identified, and spatially connected voxels above this threshold were grouped into 3D objects; only components with a minimum volume of more than four pixels were retained and assigned as peptide clusters. The automated algorithm can be accessed via https://gitlab.xlim.fr/asali_rgd/rgd_peptide_clustering_confocal.

For each hydrogel formulation, the number of such high-intensity clusters was counted per sample (n = 5–6) and used as the readout of peptide island occurrence. Statistical comparison between conditions was performed using the Mann–Whitney criterion to assess differences in cluster abundance.

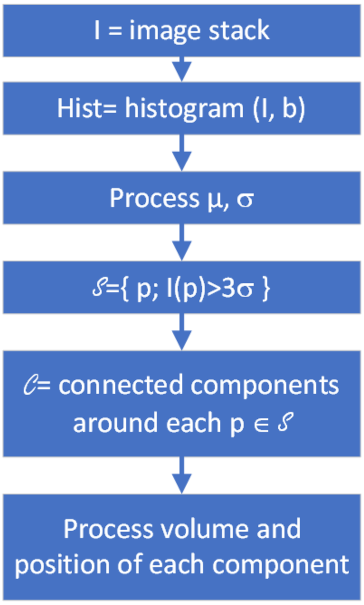

Selected NMR spectra

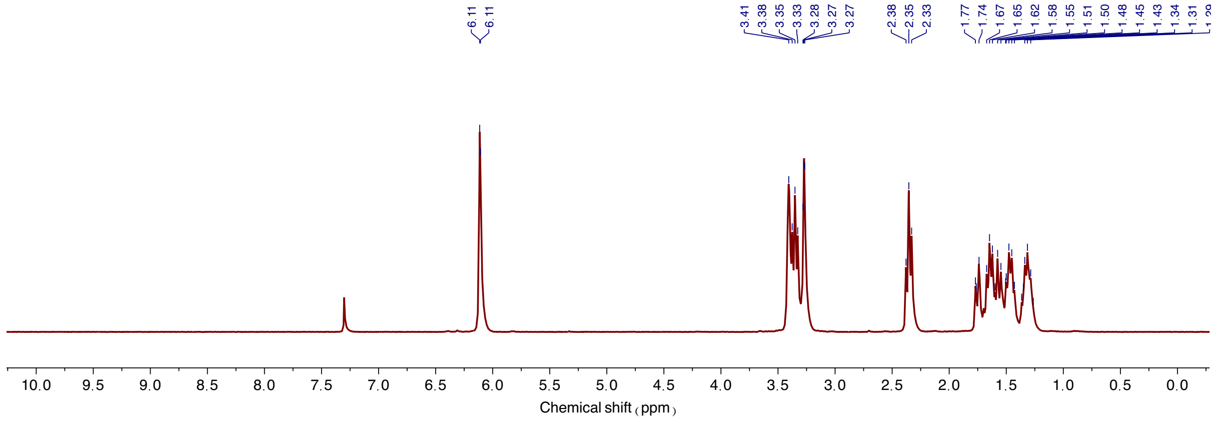

(2), $CDCl_3$

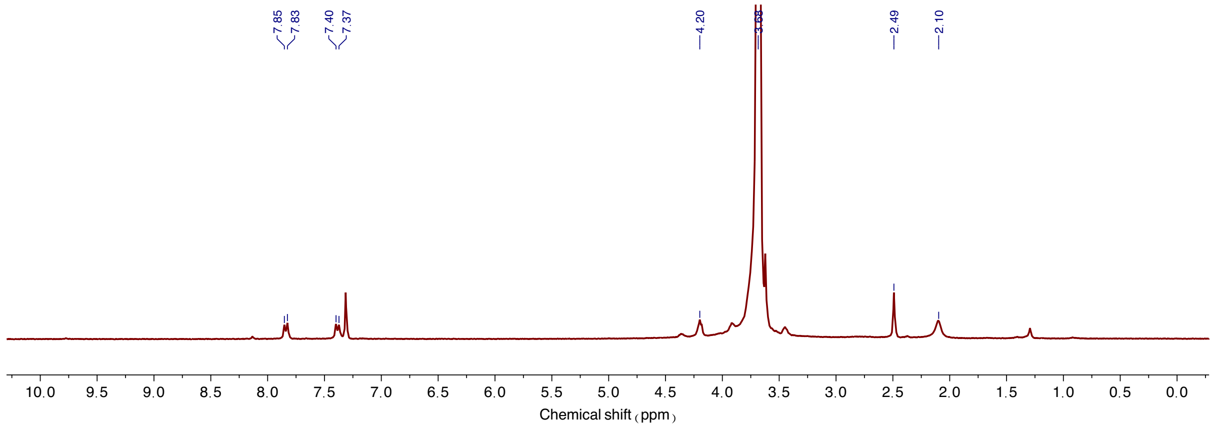

(4a), $CDCl_3$

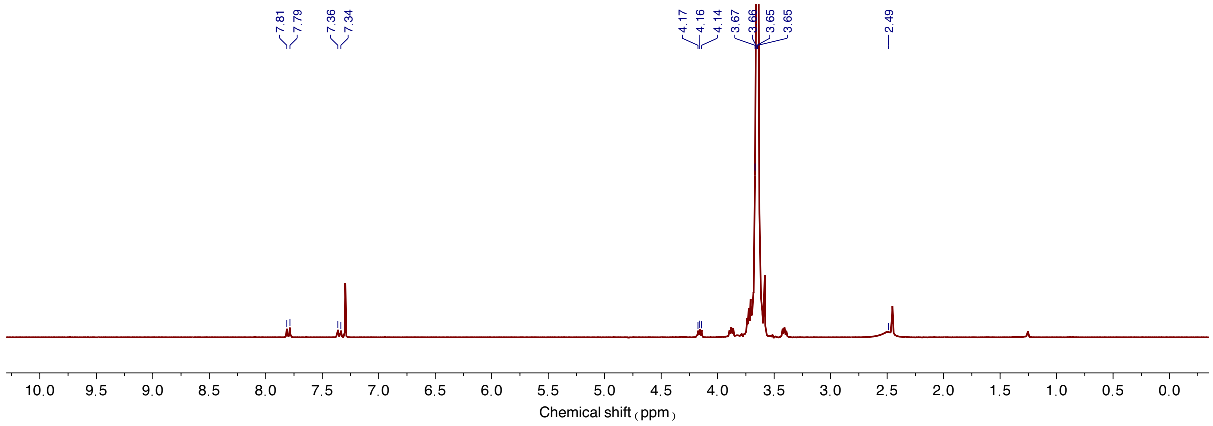

(4b), $CDCl_3$

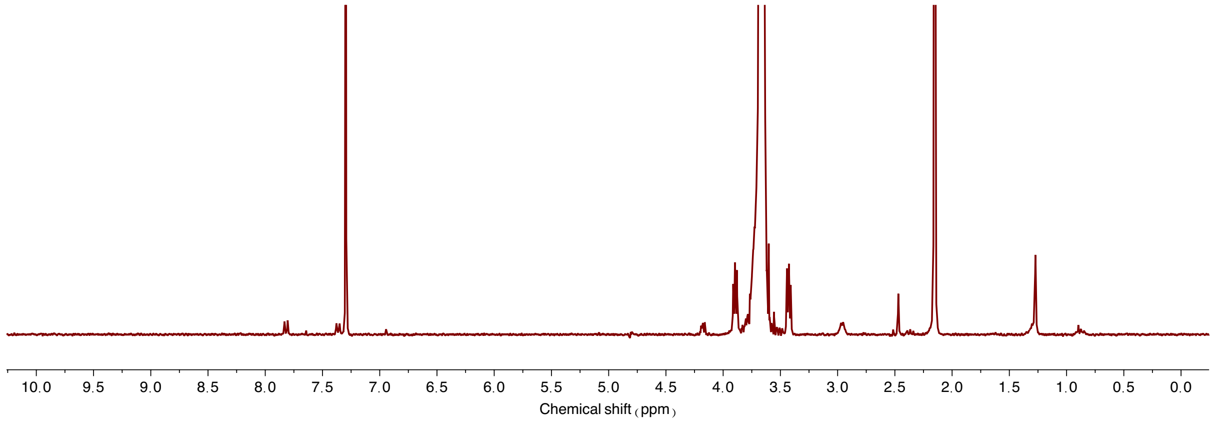

(4c), $CDCl_3$

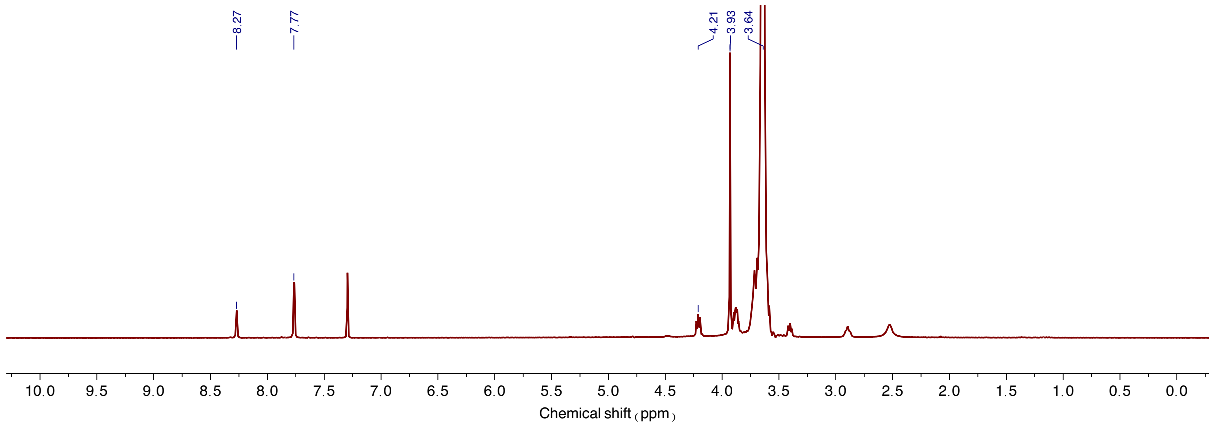

(5a), $CDCl_3$

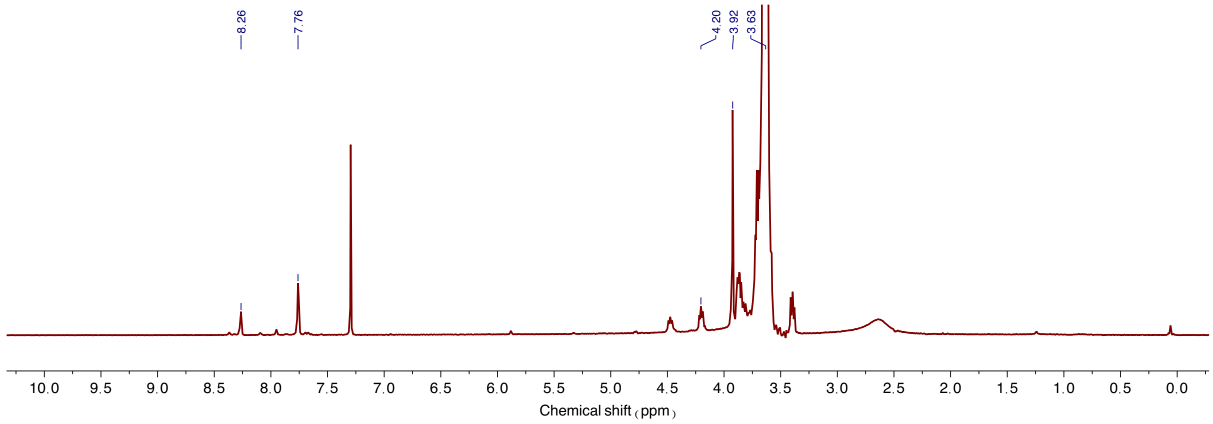

(5b), $CDCl_3$

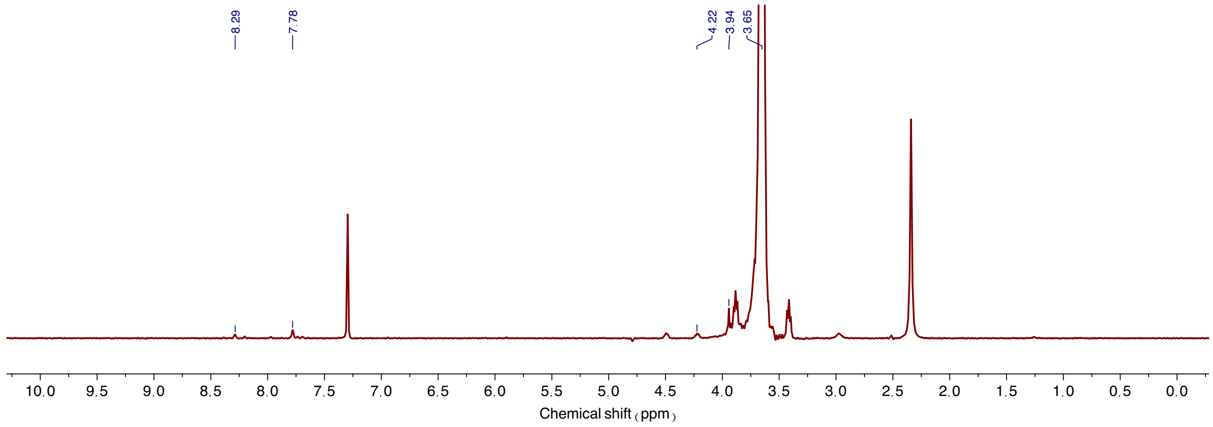

(5c), $CDCl_3$

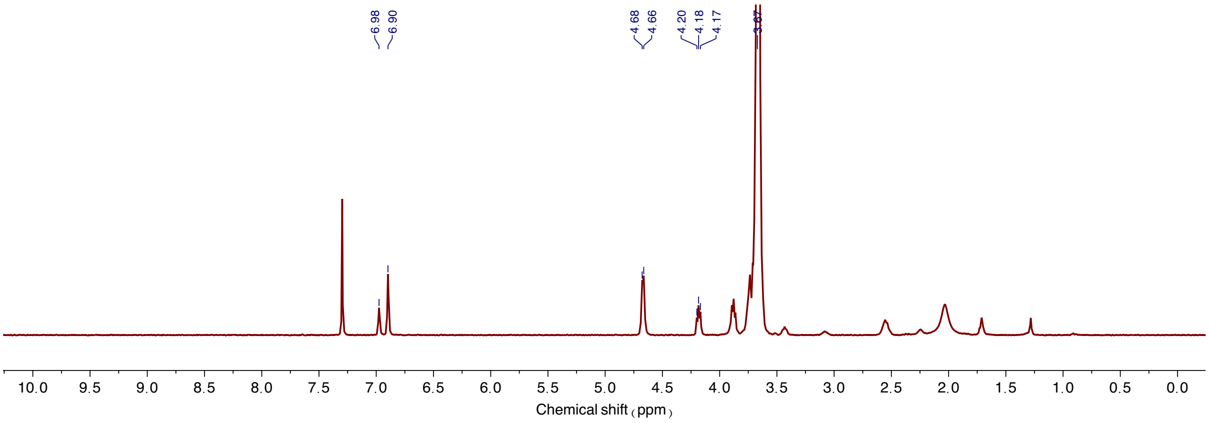

(6a), $CDCl_3$

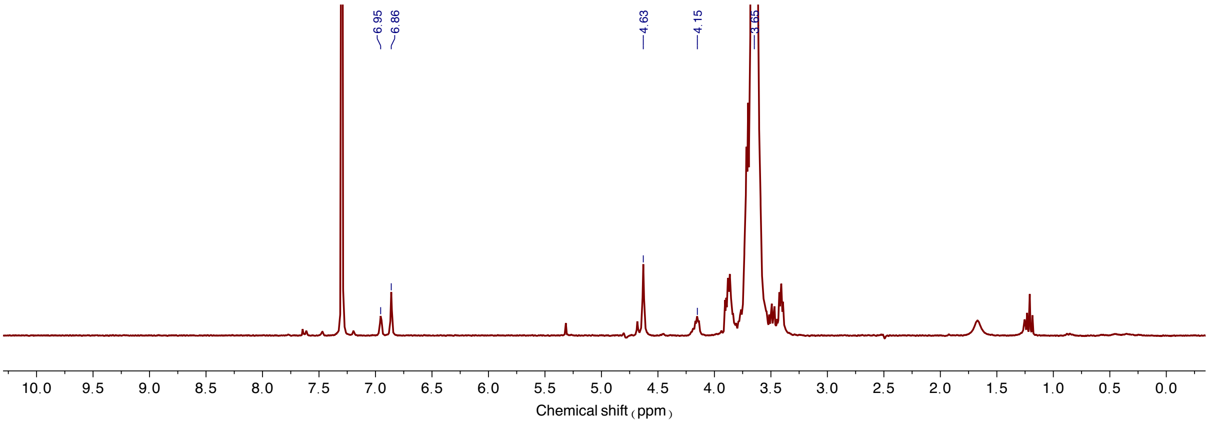

(6b), $CDCl_3$

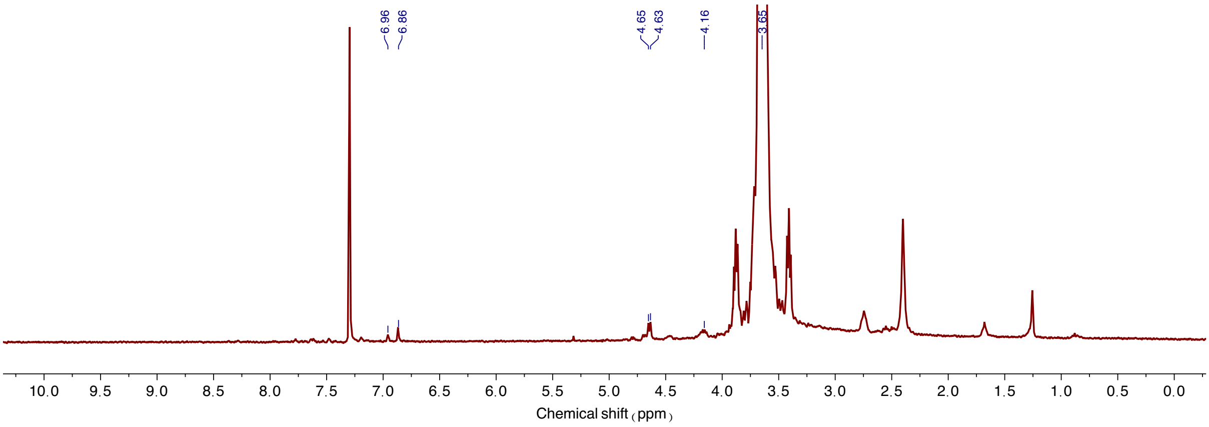

(6c), $CDCl_3$

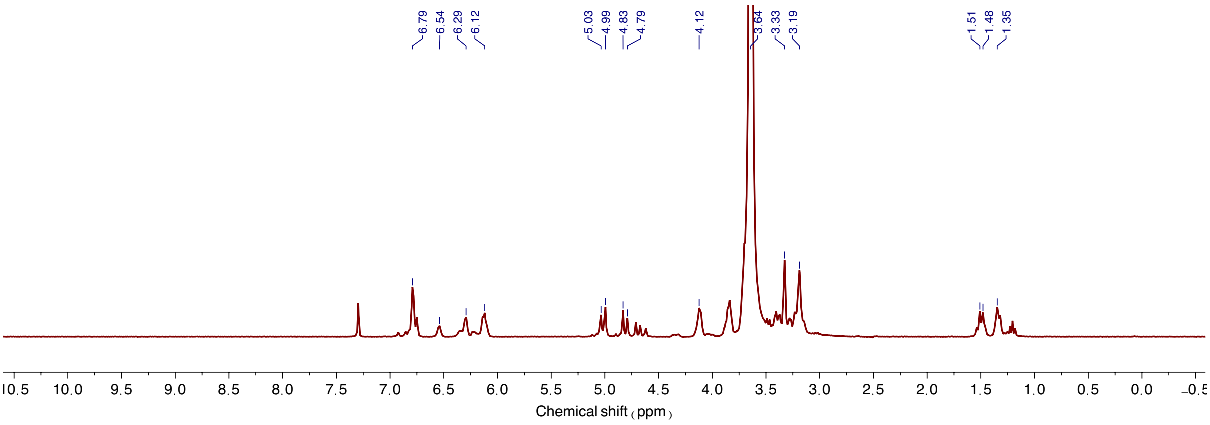

(7a), $CDCl_3$

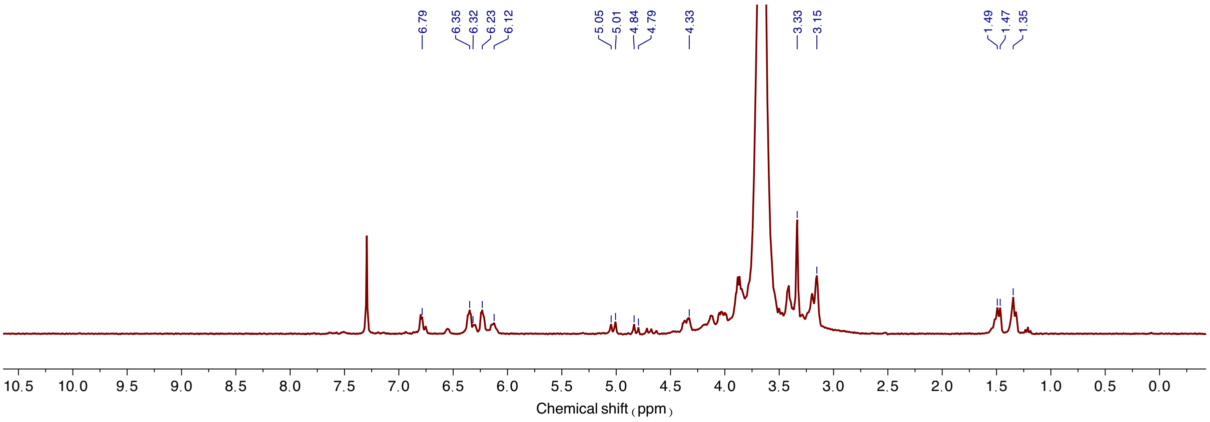

(7b), $CDCl_3$

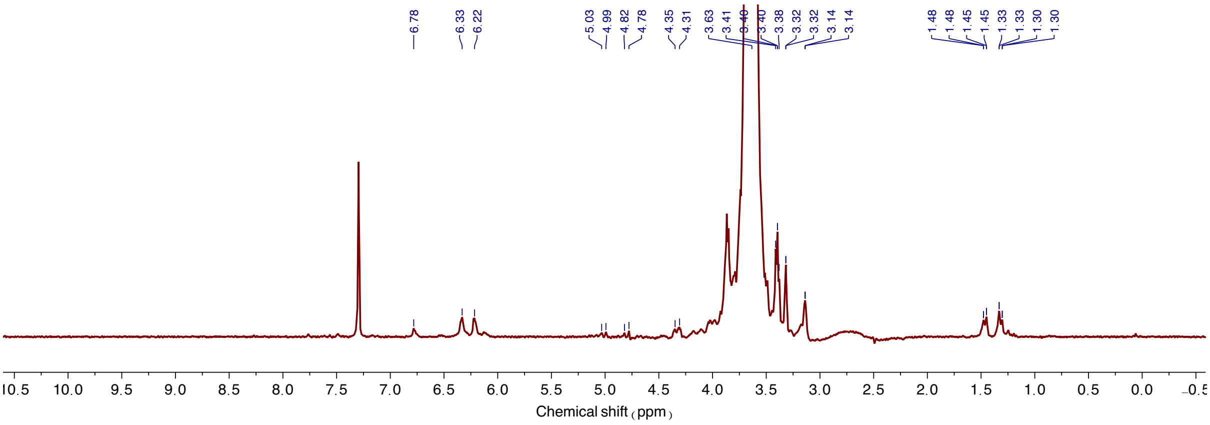

(7c), $CDCl_3$

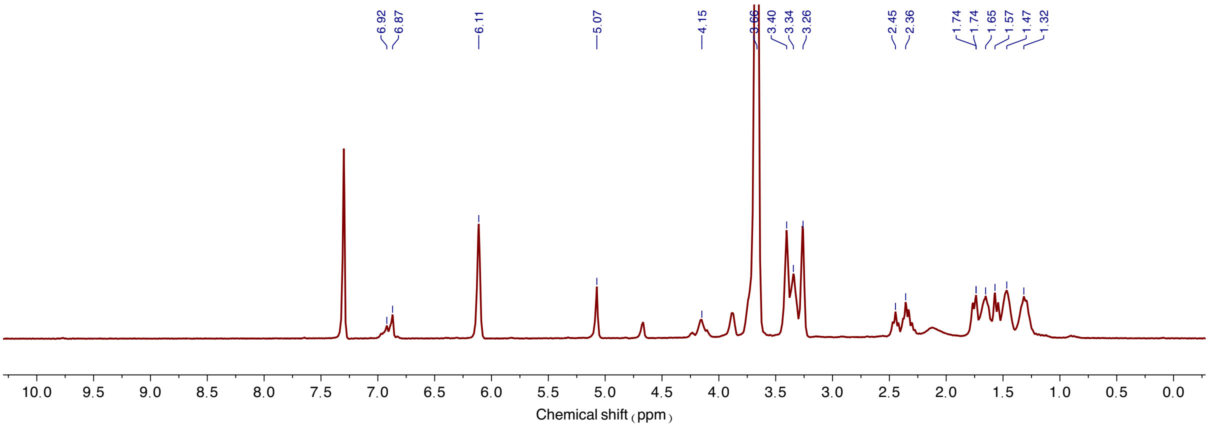

(8a), $CDCl_3$

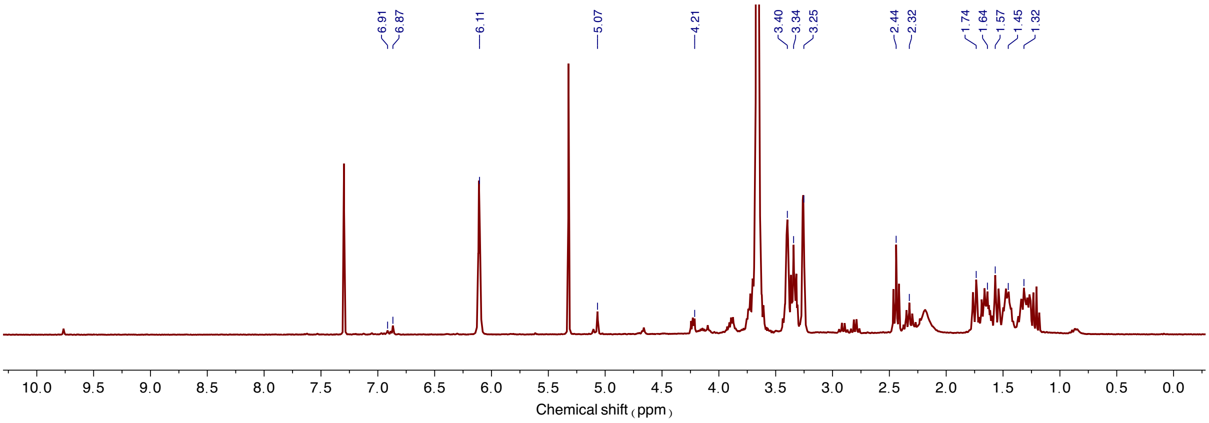

(8b), $CDCl_3$

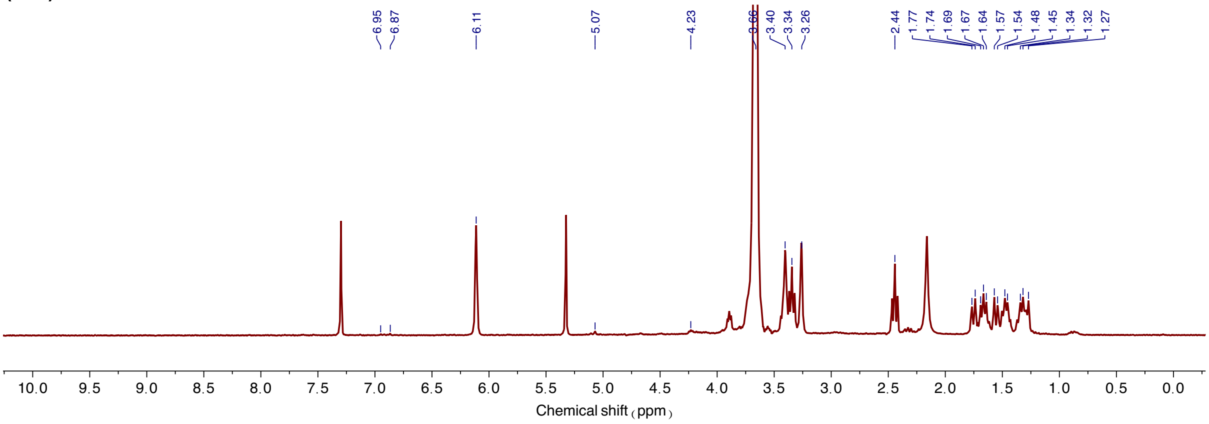

(8c), $CDCl_3$
